# The impact, mechanisms and prevention strategies of environmental endocrine disruptors on male reproductive health

**DOI:** 10.3389/fendo.2025.1573526

**Published:** 2025-10-01

**Authors:** Xiaoyan Han, Xiaolong Jin

**Affiliations:** Department of Endocrinology, Shandong Provincial Hospital Affiliated to Shandong First Medical University, Jinan, China

**Keywords:** environmental endocrine disruptors, male fertility, sperm quality, epigenetics, reproductive toxicology

## Abstract

**Background:**

Environmental endocrine disruptors (EEDs) including heavy metals, plasticizers, and persistent organic pollutants have been increasingly linked to declining male reproductive health globally. While epidemiological associations are well-established, the underlying molecular mechanisms and long-term consequences require systematic evaluation.

**Objectives:**

This review synthesizes current evidence on EED impacts on male reproductive health, focusing on molecular mechanisms, population-based evidence, transgenerational effects, and intervention strategies.

**Methods:**

We conducted comprehensive literature searches across PubMed, Web of Science, and Scopus (2019–2024) to identify peer-reviewed studies on EED reproductive toxicity, including mechanistic investigations, epidemiological studies, and intervention research.

**Results:**

EEDs disrupt male reproduction through multiple pathways: androgen and estrogen receptor interference, oxidative stress induction, mitochondrial dysfunction, and epigenetic modifications. Population studies demonstrate consistent associations between EED exposure and reduced sperm quality, with effect sizes varying by exposure level and chemical type. Animal studies provide compelling evidence for transgenerational inheritance of reproductive dysfunction through epigenetic mechanisms, though human evidence remains limited. Workplace protection measures, environmental remediation, and policy interventions show promise but require broader implementation.

**Conclusions:**

EEDs pose significant threats to male reproductive health through complex, interconnected mechanisms. While substantial progress has been made in understanding these effects, critical gaps remain in mixture toxicology, low-dose effects, and transgenerational impacts in humans. Enhanced biomonitoring, mechanism-based interventions, and strengthened regulatory frameworks are essential for protecting current and future reproductive health.

## 1 Introduction

The unprecedented pace of industrialization and urbanization over recent decades has introduced a vast array of chemical contaminants into our environment, among which environmental endocrine disruptors (EEDs) have emerged as a particularly concerning threat to human reproductive health (1). These synthetic and naturally occurring substances interfere with normal hormonal signaling pathways, with mounting evidence suggesting they play a pivotal role in the global decline of male fertility. Contemporary epidemiological research has documented an alarming 50% reduction in sperm concentration over the past four decades–a temporal trend that closely parallels the exponential increase in EED production and environmental distribution (2). While the association between environmental contamination and reproductive dysfunction has long been suspected, establishing definitive mechanistic links between specific EED exposures and male reproductive impairment remains a complex scientific challenge (3).

### 1.1 Sources and classification of EEDs

Environmental endocrine disruptors encompass a remarkably diverse array of chemical compounds that can be systematically categorized into three distinct groups based on their origin, chemical properties, and environmental behavior:

Heavy Metals and Metalloids represent one of the most persistent categories of reproductive toxicants. Lead and cadmium, for instance, demonstrate extraordinary environmental persistence with biological half-lives extending 20–30 years in human tissues. These metals primarily infiltrate human systems through contaminated drinking water, food chain bioaccumulation, and occupational exposure in industrial settings.

Synthetic Organic Compounds constitute the largest and most ubiquitous category of EEDs. Bisphenol A (BPA) and phthalates, omnipresent in consumer products ranging from food packaging to personal care items, lead to widespread human exposure, with typical daily intake levels of 0.1-4 µg/kg body weight for BPA and 1-20 µg/kg body weight for phthalates. These compounds are particularly concerning due to their widespread distribution and continuous low-level exposure patterns.

Persistent Organic Pollutants (POPs), including dioxins and polychlorinated biphenyls (PCBs), represent the most environmentally stable class of EEDs. Their exceptional chemical stability allows environmental persistence exceeding two decades, leading to progressive bioaccumulation through food webs and preferential storage in lipid-rich tissues (4).

Human exposure to these compounds occurs through multiple pathways—ingestion, inhalation, and dermal absorption–with subsequent accumulation in adipose tissue, hepatic systems, and critically, reproductive organs (5). Biomonitoring studies consistently reveal that the majority of human biological samples contain detectable concentrations of multiple EEDs, indicating widespread population exposure across diverse demographic groups (6).

### 1.2 Key research challenges

Despite substantial advances in understanding individual EED toxicity profiles, several fundamental knowledge gaps continue to impede comprehensive risk assessment:

Complex Mixture Effects Remain Poorly Understood While toxicological studies have extensively characterized individual EED effects, real-world human exposure invariably involves simultaneous contact with multiple compounds. Research on mixture effects involving multiple EEDs remains an emerging field, with most studies focusing on single-chemical exposures despite the reality of multi-chemical environmental exposure, limiting our understanding of cumulative health risks (7).

Transgenerational Impacts Lack Adequate Human Documentation Animal studies consistently demonstrate that EED exposure can induce heritable epigenetic modifications affecting offspring fertility across multiple generations. However, human epidemiological evidence for transgenerational effects remains limited, primarily due to the extended timeframes required for multigenerational studies and the practical challenges of maintaining long-term cohorts across decades (8).

Low-Dose Chronic Exposure Effects Present Assessment Challenges EEDs frequently exhibit non-monotonic dose-response (NMDR) patterns, where low-dose chronic exposure may produce more pronounced biological effects than acute high-dose exposure. This phenomenon is further complicated by reported no observed adverse effect levels (NOAELs) that vary by 2–3 orders of magnitude across studies, likely reflecting differences in metabolic processing, genetic susceptibility, and methodological approaches (9).

### 1.3 Objectives of this review

This comprehensive review synthesizes current evidence (2019–2024) regarding EED-mediated reproductive toxicity in males, with particular emphasis on:

Mechanistic Understanding: Elucidating the molecular pathways through which EEDs disrupt male reproductive function, including oxidative stress cascades, endocrine signaling interference, and epigenetic modifications.

Real-World Exposure Assessment: Evaluating the synergistic effects of multiple EED exposure to better reflect actual human environmental conditions and improve risk prediction accuracy.

Early Detection Strategies: Developing epigenetic biomarker-based early warning systems for identifying individuals at risk of EED-induced reproductive impairment before clinical manifestation.

Evidence-Based Interventions: Identifying and validating protective strategies and therapeutic interventions capable of mitigating EED-induced reproductive damage.

Through systematic evaluation of EED impacts on male fertility and transgenerational health, this review aims to address critical knowledge gaps, inform evidence-based environmental and public health policies, and facilitate development of targeted clinical intervention strategies.

## 2 Types and sources of environmental endocrine disruptors

**Figure 1:**
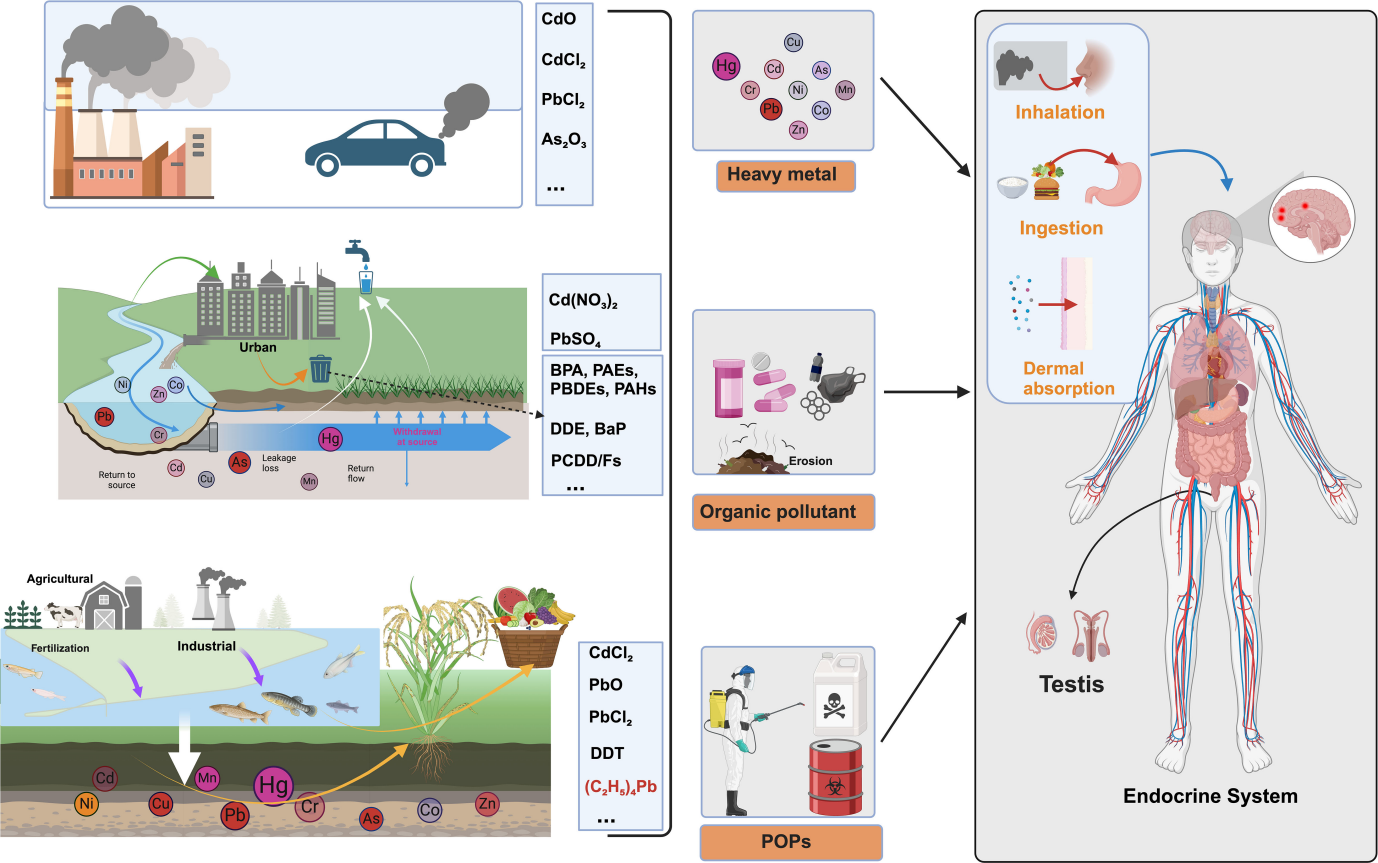
Classification and exposure pathways of environmental endocrine disruptors (EEDs). This comprehensive schematic illustrates the complex environmental sources, chemical classification, and human exposure pathways of environmental endocrine disruptors. Environmental Sources: The diagram depicts major contamination sources including industrial emissions (factories with smokestacks), vehicular exhaust, urban runoff from metropolitan areas, agricultural fertilization, and industrial discharge into water systems. Three Primary EED Categories: Heavy metals are represented by specific compounds including cadmium oxide (CdO), cadmium chloride (CdCl_2_), lead chloride (PbCl_2_), and arsenic trioxide (As_2_O_3_), along with elements Hg, Cd, As, Cr, Pb, Co, Zn, and Mn that demonstrate exceptional biological persistence (20–30 year half-lives). Organic pollutants encompass cadmium nitrate [Cd(NO_3_)_2_], lead sulfate (PbSO_4_), bisphenol A (BPA), phthalates (PAEs), polybrominated diphenyl ethers (PBDEs), polycyclic aromatic hydrocarbons (PAHs), dichlorodiphenyldichloroethylene (DDE), benzo[a]pyrene (BaP), and polychlorinated dibenzo-p-dioxins/furans (PCDD/Fs), commonly found in consumer products and plastic materials. Persistent organic pollutants (POPs) include cadmium chloride (CdCl_2_), lead oxide (PbO), lead chloride (PbCl_2_), dichlorodiphenyltrichloroethane (DDT), and tetraphenyllead [(C_6_;H_5_)_4_Pb], characterized by environmental persistence exceeding decades. Human Exposure Pathways: The anatomical diagram demonstrates three critical exposure routes—inhalation of contaminated air particles, ingestion of contaminated food and water, and dermal absorption through direct skin contact—with EEDs subsequently targeting the endocrine system and accumulating specifically in testicular tissues, ultimately disrupting male reproductive function.

Environmental endocrine disruptors represent a heterogeneous collection of chemical substances that fundamentally alter hormone signaling pathways, thereby compromising endocrine homeostasis and reproductive function. These compounds originate from numerous industrial, agricultural, and consumer sources, with their environmental persistence stemming from inherent chemical stability. Understanding their classification schemes, exposure pathways, and health implications has become crucial given their pervasive environmental distribution (**Figure 1**) (10).

### 2.1 Systematic classification of environmental endocrine disruptors

The complexity of EED classification reflects the diverse origins and chemical properties of these compounds. Based on structural characteristics and environmental fate, EEDs can be organized into three primary categories: metallic and metalloid compounds, synthetic organic compounds, and persistent organic pollutants (POPs).

#### 2.1.1 Heavy metal elements

Heavy metals and their derivatives pose significant reproductive health risks due to their propensity for bioaccumulation and cellular disruption. Their toxicity profiles reflect both their chemical reactivity and remarkable biological persistence.

##### 2.1.1.1 Heavy metals

Among the most concerning reproductive toxicants, cadmium demonstrates exceptional persistence with a biological half-life spanning 20–30 years. This metal preferentially accumulates in renal and reproductive tissues, where it compromises the blood-testis barrier integrity and substantially impairs sperm quality parameters (11). Lead exposure presents equally serious concerns–when blood lead concentrations exceed 10 μg/dL, significant sperm DNA damage becomes evident, with seminal lead accumulation reaching concentrations of 3.2 ± 0.8 μg/dL (12). Arsenic toxicity manifests primarily through its metabolite monomethylarsonic acid (DMA), which disrupts testosterone biosynthesis pathways when occupational exposure levels surpass 0.01 mg/m³ (13).

##### 2.1.1.2 Metal oxides

The emergence of engineered nanoparticles has introduced novel exposure scenarios. zinc oxide nanoparticles (ZnO-NPs) readily penetrate the blood-testis barrier, triggering inflammatory cascades that substantially impair sperm motility (14). Similarly, titanium dioxide exposure induces excessive reactive oxygen species (ROS) production, resulting in sperm membrane damage with an ED50 of 150 mg/kg (15).

#### 2.1.2 Metal oxide nanoparticles

This category encompasses the most widespread EEDs in modern environments, primarily due to their extensive industrial applications and consumer product integration. These compounds disrupt endocrine function through direct receptor binding and metabolic pathway interference.

##### 2.1.2.1 Plastic monomers and additives

Bisphenol A remains ubiquitous in plastic manufacturing despite established tolerable daily intake limits of 50 μg/kg. Its endocrine-disrupting potential stems from high-affinity binding to estrogen receptors, fundamentally altering hormonal balance (16). Phthalates, particularly di(2-ethylhexyl) phthalate (DEHP), are routinely detected in seminal plasma at concentrations of 0.77-1.85 μg/mL, with documented associations to reduced sperm concentration and motility (17).

##### 2.1.2.2 Flame retardants

Polybrominated diphenyl ethers (PBDEs) exhibit remarkable biological persistence, with half-lives extending 3–7 years and high octanol-water partition coefficients (log Kow = 6.5-8.4), facilitating extensive bioaccumulation. These compounds interfere with both thyroid and androgen hormone signaling pathways (18). Hexabromocyclododecane (HBCD) demonstrates even greater bioaccumulative potential with bioaccumulation factors exceeding 5000, significantly affecting testicular development and spermatogenesis (19).

#### 2.1.3 Persistent organic pollutants

POPs represent the most environmentally stable EEDs, persisting for decades while progressively bioaccumulating through food webs, creating long-term health risks that extend beyond direct exposure.

##### 2.1.3.1 Industrial sources

Polychlorinated biphenyls exemplify POPs characteristics with half-lives of 8–15 years and extraordinary lipophilicity (bioconcentration factors >10^5^). This combination ensures extensive adipose tissue accumulation and sustained reproductive system effects (20). Dioxins, including polychlorinated dibenzo-p-dioxins and furans (PCDD/Fs), demonstrate extreme toxicity with toxic equivalence factors ranging from 0.0001 to 1. These compounds severely disrupt androgen synthesis and compromise sperm development (21).

##### 2.1.3.2 Agricultural sources

Despite widespread regulatory restrictions, organochlorine pesticides like DDT continue to pose reproductive health risks due to environmental persistence. DDT and its primary metabolite DDE remain detectable in biological samples decades after application cessation, continuing to disrupt testosterone synthesis pathways (22). Polycyclic aromatic hydrocarbons (PAHs), generated through combustion processes, contribute additional toxicity. Benzo[a]pyrene, with an ED50 of 150 μg/kg, induces direct DNA damage in sperm cells (**Table 1**) (23).

**Table 1:** Classification and mechanisms of damage of environmental endocrine disruptors (EEDs).

Category	Substance	Environmental persistence/exposure limit	International standard/threshold	Mechanism of damage
Metallic and Metalloid Compounds	Cadmium (Cd)	Half-life: 20–30 years	WHO tolerable intake: 7 μg/kg/week	Impairs sperm quality, induces oxidative stress
	Lead (Pb)	Blood lead >10 μg/dL	CDC reference level: 3.5 μg/dL (children), 5 μg/dL (adults, action level)	Causes DNA damage, reduces fertility
	Arsenic (As)	Occupational limit: 0.01 mg/m³	ACGIH TLV-TWA: 0.01 mg/m³	Disrupts testosterone synthesis, causes reproductive disorders
Synthetic Organic Compounds	BPA	Permissible intake: 50 μg/kg/day (USA), 0.2 ng/kg/day (EU, 2023 update)	EFSA/US EPA limit	Disrupts estrogen receptors (ER), reduces sperm quality
	Phthalates (PAEs)	Seminal plasma: 0.77–1.85 µg/mL	EFSA daily intake limit: 50 µg/kg	Reduces sperm concentration and motility
Persistent Organic Pollutants (POPs)	Polychlorinated Biphenyls (PCBs)	Half-life: 8–15 yr, BCF >10^5	WHO TEF range: 0.0001–1	Cumulative toxicity impacting reproductive system
	Dioxins (PCDD/Fs)	TEF range: 0.0001–1	WHO tolerable intake: 1–4 pg TEQ/kg/week	Disrupts testosterone synthesis and spermatogenesis

### 2.2 Major exposure pathways and representative cases

Human EED exposure occurs through multiple routes, with patterns varying significantly based on occupational activities, environmental conditions, and consumer behaviors.

#### 2.2.1 Occupational exposure

Workplace environments often represent the highest EED exposure scenarios, particularly in electronics manufacturing and agricultural sectors.

##### 2.2.1.1 Electronics manufacturing industry

A comprehensive meta-analysis conducted in 2023 examined lead exposure among semiconductor workers, revealing significant reproductive health impacts. Exposed workers demonstrated blood lead levels of 3.2 ± 0.8 μg/dL compared to 1.2 ± 0.3 μg/dL in control populations. This occupational exposure resulted in measurable decreases in sperm motility (↓1.31%, 95% CI -2.33 to -0.30) and vitality (↓2.18%, 95% CI -3.92 to -0.45) (24).

##### 2.2.1.2 Agricultural industry

Cross-sectional studies of pesticide applicators compared to unexposed controls revealed concerning exposure patterns. Approximately 62% of agricultural workers exhibited elevated urinary organophosphate metabolites, corresponding to a 68% increase in sperm malformation rates (25).

#### 2.2.2 Environmental exposure

Regional environmental contamination creates population-wide exposure risks with documented reproductive health consequences.

##### 2.2.2.1 Water contamination studies

Research conducted in the Lower Yangtze River region (2022–2023) documented dioxin concentrations of 4.8 pg TEQ/g, exceeding WHO safety guidelines by 20%. Male residents in affected areas demonstrated a 28% reduction in sperm concentration, suggesting direct correlation between environmental dioxin exposure and reproductive function (26).

##### 2.2.2.2 Air quality impacts

Proximity studies show that residents living within several kilometers of major industrial facilities experience elevated PAH exposure levels compared to background populations. This exposure level corresponds to an 1.8-fold increase in male infertility risk, likely attributable to oxidative stress-induced DNA damage (27).

#### 2.2.3 Daily consumer contact

Routine consumer product use creates widespread, low-level EED exposure across populations.

##### 2.2.3.1 Food packaging contamination

A 2023 market survey examining 500 baby bottles revealed BPA migration levels reaching 0.75 µg/L, with 15% of samples exceeding established safety thresholds. Elevated BPA exposure in these products correlated with altered hormonal profiles related to pubertal development in infants (28).

##### 2.2.3.2 Household product exposure

Analysis of indoor air and dust samples (n=50) revealed that 96% and 98% of households contained detectable levels of TDCPP and TPP flame retardants, respectively. Chronic exposure to these compounds was associated with a 19% reduction in sperm concentration (29).

### 2.3 Emerging methods for exposure assessment

Technological advances have significantly enhanced EED detection capabilities and risk assessment precision.

#### 2.3.1 Biomarker monitoring

Contemporary biomonitoring approaches utilize sophisticated analytical techniques to quantify EED exposure and biological effects. Urinary metabolomics now enables precise measurement of monoester phthalates and BPA glucuronide conjugates, providing accurate assessments of recent exposure burden (30). Serum proteomics approaches evaluate sperm membrane protein integrity and oxidative stress markers, offering insights into biological effect mechanisms (31).

#### 2.3.2 Environmental monitoring technologies

Innovative monitoring technologies have revolutionized exposure assessment capabilities. Passive sampling devices enable cumulative exposure evaluation across temporal and spatial scales, providing integrated exposure profiles (32). High-throughput screening platforms rapidly detect multiple pollutants simultaneously while assessing their biological effects, significantly improving risk characterization efficiency (33).

## 3 Molecular mechanisms of environmental endocrine disruptors’ impact on male reproductive health

**Figure 2:**
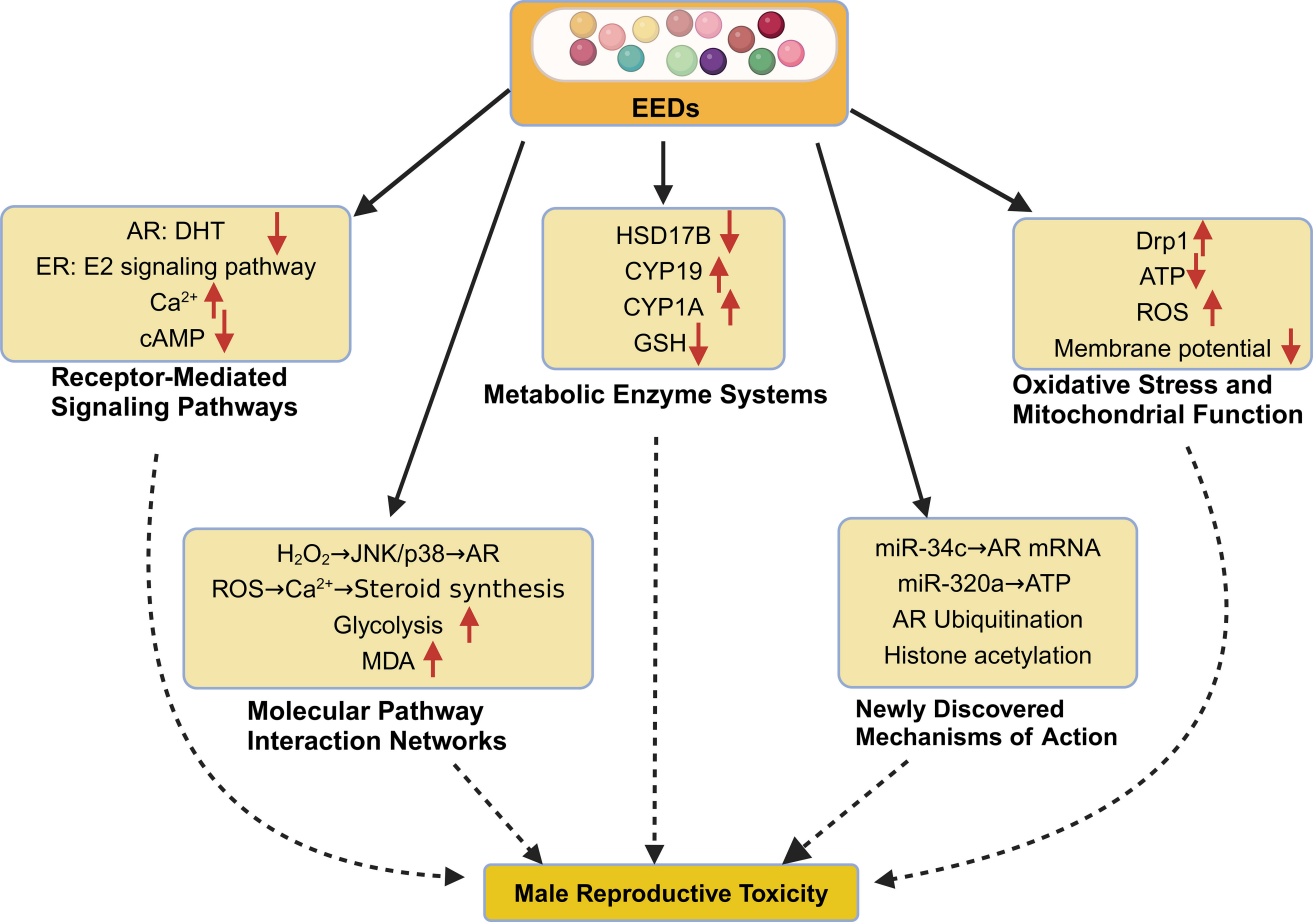
Molecular mechanisms of EED-induced reproductive toxicity in males. This schematic illustrates the complex, interconnected molecular pathways through which environmental endocrine disruptors compromise male reproductive function. 1) Receptor-Mediated Signaling Disruption: EEDs interfere with nuclear hormone receptors, including androgen receptor (AR) antagonism (85% reduction in ARE activity, Ki = 4.3×10^−6^ M for BPA) and estrogen receptor (ER) modulation (ERα EC50 = 0.8 μM, ERβ EC50 = 1.2 μM for PCBs). Membrane signaling alterations include calcium homeostasis disruption (3.2-fold Ca^2+^ elevation) and cAMP pathway suppression (45% reduction). 2) Metabolic Enzyme System Dysregulation: Critical steroidogenic enzymes undergo inhibition, including 17β-hydroxysteroid dehydrogenase (HSD17B) suppression (IC50 = 2.2 μM for cadmium, 85% testosterone synthesis reduction) and aromatase (CYP19) upregulation (2.3-fold mRNA increase, 56% activity enhancement). Detoxification systems show impairment with glutathione (GSH) depletion to 35% of normal levels. 3) Oxidative Stress and Mitochondrial Dysfunction: Enhanced reactive oxygen species (ROS) production (3.4-fold increase) accompanies mitochondrial dysfunction, including 52% Complex I activity reduction, 67% ATP production decrease, and 45% membrane potential decline. 4) Molecular Pathway Integration: Oxidative stress cascades intersect with disrupted signaling pathways, affecting glycolytic metabolism (2.1-fold increase) and steroid synthesis coordination. 5) Emerging Epigenetic Mechanisms: Novel regulatory disruptions include microRNA dysregulation (miR-34c targeting AR mRNA, miR-320a affecting mitochondrial proteins), androgen receptor ubiquitination-mediated degradation, and histone modification alterations (H3K27ac, H3K4me3 dysregulation).

Environmental endocrine disruptors exert their deleterious effects on male reproductive physiology through complex, interconnected molecular pathways that fundamentally disrupt normal cellular processes. These mechanisms encompass hormonal signaling interference, metabolic pathway disruption, oxidative stress induction, and epigenetic modifications–each contributing to impaired spermatogenesis, compromised sperm function, and broader endocrine dysfunction. Understanding these molecular mechanisms provides crucial insights into how EEDs translate environmental exposure into reproductive pathology (**Figure 2**).

### 3.1 Receptor-mediated signaling pathways

EEDs primarily compromise reproductive function by interfering with nuclear hormone receptor systems and membrane-associated signaling cascades that orchestrate normal reproductive physiology.

#### 3.1.1 Nuclear receptor signaling disruption

##### 3.1.1.1 Androgen receptor antagonism

Bisphenol A exhibits androgenic antagonism, functioning as a competitive antagonist that blocks androgen receptor binding and subsequently reduces ARE-mediated gene transcription, with documented effects on androgen-dependent gene expression. This interference effectively blocks androgen-dependent gene expression essential for normal male reproductive development (34). The disruption extends beyond direct receptor binding-EED exposure significantly impairs recruitment of critical coactivators such as steroid receptor coactivator-1 (SRC-1), substantially weakening AR transcriptional capacity (35).

##### 3.1.1.2 Estrogen receptor modulation

Polychlorinated biphenyls exhibit differential binding affinities for estrogen receptor subtypes, with ERα demonstrating an EC50 of 0.8 μM and ERβ showing an EC50 of 1.2 μM. This selective binding initiates both genomic and non-genomic estrogenic responses that disrupt normal hormonal balance (36). Additionally, PCB exposure upregulates GPR30 signaling pathways by 2.3-fold, amplifying non-genomic estrogen responses and further complicating hormonal homeostasis (37).

#### 3.1.2 Membrane receptor and calcium signaling dysregulation

##### 3.1.2.1 Calcium homeostasis disruption

EED exposure significantly disrupts intracellular calcium homeostasis, leading to aberrant Ca^2+^ signaling patterns that compromise sperm function. This dysregulation severely impairs sperm motility and acrosome reaction capacity-critical processes for successful fertilization (38). The mechanism involves altered calcium channel activity, particularly affecting voltage-gated calcium channels (CaVs) and store-operated calcium entry pathways, leading to either excessive calcium influx or impaired calcium release from intracellular stores, ultimately resulting in cellular dysfunction and reduced fertilization potential (39).

##### 3.1.2.2 G-protein coupled receptor pathway suppression

EEDs systematically suppress GPCR-mediated signaling cascades essential for steroidogenesis and germ cell proliferation. This suppression manifests as a 45% reduction in cyclic adenosine monophosphate (cAMP) levels and 53% inhibition of protein kinase A (PKA) activity, effectively disrupting the molecular machinery required for normal testicular function (40).

### 3.2 Metabolic enzyme regulation and steroidogenesis

EED interference with critical enzymatic pathways responsible for testosterone biosynthesis and xenobiotic detoxification creates profound hormonal imbalances that compromise reproductive function.

#### 3.2.1 Steroidogenic enzyme inhibition

##### 3.2.1.1 17β-hydroxysteroid dehydrogenase (HSD17B) suppression

Cadmium exposure directly inhibits HSD17B enzymatic activity with an IC50 of 2.2 μM, resulting in an 85% reduction in testosterone synthesis capacity. This enzyme is crucial for the final step in testosterone biosynthesis, making its inhibition particularly devastating for androgen production (41).

##### 3.2.1.2 Aromatase (CYP19) dysregulation

EED exposure paradoxically upregulates CYP19 mRNA expression by 2.3-fold while simultaneously increasing catalytic activity (Vmax) by 56%. This enhancement of testosterone-to-estradiol conversion disrupts the androgen-to-estrogen balance essential for normal male reproductive function (42).

#### 3.2.2 Detoxification pathway impairment

##### 3.2.2.1 Cytochrome P450 system dysfunction

EED exposure creates a dual dysfunction within the cytochrome P450 system. CYP1A1 activity increases 4.6-fold, leading to excessive free radical production and subsequent DNA damage, while CYP3A4 expression decreases by 68%, severely impairing steroid hormone metabolism (43, 44).

##### 3.2.2.2 Glutathione system compromise

The cellular antioxidant defense system suffers significant impairment, with GSH levels declining to 35% of normal values, substantially weakening antioxidant defenses. Simultaneously, glutathione-S-transferase (GST) activity decreases by 72%, reducing the cellular capacity to clear toxic metabolites (45, 46).

### 3.3 Oxidative stress and mitochondrial dysfunction

EED-induced oxidative stress represents a central mechanism underlying reproductive toxicity, with mitochondrial dysfunction serving as both cause and consequence of excessive reactive oxygen species production.

#### 3.3.1 Mitochondrial dynamics disruption

The cellular machinery governing mitochondrial fusion and fission becomes severely dysregulated following EED exposure. Dynamin-related protein 1 (Drp1) phosphorylation increases 3.1-fold, promoting excessive mitochondrial fission and compromising sperm motility (47). Concurrently, mitofusin 2 (Mfn2) expression decreases by 65%, leading to mitochondrial fragmentation and metabolic inefficiency (48). These mitochondrial alterations are accompanied by changes in nuclear-encoded mitochondrial gene expression, suggesting epigenetic regulation of cellular energy metabolism pathways (49).

#### 3.3.2 Energy metabolism impairment

##### 3.3.2.1 Electron transport chain dysfunction

EED exposure severely compromises mitochondrial energy production through direct effects on the electron transport chain. Complex I dysfunction results in substantially reduced ATP production, creating an energy deficit that directly compromises sperm motility and cellular function (50). This energetic crisis is compounded by a 45% decline in mitochondrial membrane potential while ROS production increases 3.4-fold, creating a cascade of cellular damage (51).

### 3.4 Molecular pathway interaction networks

The reproductive toxicity induced by EEDs results from complex crosstalk among oxidative stress responses, metabolic disruption, and immune system activation rather than isolated pathway effects.

#### 3.4.1 Oxidative stress-endocrine axis integration

ROS accumulation triggers activation of stress-activated protein kinases (JNK/p38 pathway), which subsequently inhibits AR transcriptional activity, creating a cascade that disrupts testosterone synthesis (52). Additionally, mitochondrial ROS production alters calcium signaling dynamics, further dysregulating steroidogenesis and affecting sperm development (53).

#### 3.4.2 Metabolism-immune-epigenetic crosstalk

Inflammatory mediators including TNF-α and NF-κB activation modulate AR signaling capacity while IL-6 triggers DNA methylation changes that affect spermatogenesis (54). The metabolic reprogramming includes a 2.1-fold increase in glycolytic activity (Warburg effect) accompanied by 4.3-fold elevation in malondialdehyde (MDA) levels, indicating oxidative stress-mediated metabolic reorganization (55).

### 3.5 Emerging epigenetic mechanisms

Recent investigations have revealed the critical role of non-coding RNAs and chromatin modifications in mediating EED-induced reproductive toxicity, providing new insights into both immediate and transgenerational effects.

#### 3.5.1 Non-coding RNA regulatory networks

##### 3.5.1.1 MicroRNA dysregulation

Specific miRNA species serve as critical mediators of EED toxicity. miR-34c directly targets AR mRNA, inhibiting androgen receptor translation and compromising sperm maturation processes (56). Similarly, miR-320a targets essential mitochondrial proteins including Cox8a and ATP5b, reducing oxidative phosphorylation efficiency and impairing sperm motility (57).

##### 3.5.1.2 Long non-coding RNA alterations

NEAT1 undergoes upregulation following BPA exposure, influencing spermatogonial stem cell fate determination through miR-148a-3p sponging mechanisms (58). The H19 lncRNA modulates DNA methylation patterns in germ cells, potentially contributing to transgenerational inheritance of reproductive dysfunction (59).

#### 3.5.2 Protein modifications and chromatin remodeling

##### 3.5.2.1 Post-translational modifications

EED exposure promotes androgen receptor protein ubiquitination, accelerating AR degradation and reducing transcriptional activity (60). Critical histone modifications including H3K27ac and H3K4me3 undergo dysregulation, substantially altering reproductive gene expression patterns (61). Additionally, SUMOylation of transcription factors impairs their nuclear localization, further disrupting hormonal signaling cascades (62, 63).

These diverse molecular mechanisms collectively demonstrate that EED-induced reproductive toxicity results from systematic disruption of multiple, interconnected biological pathways rather than simple receptor binding events.

## 4 Evidence of environmental endocrine disruptors’ impact on male fertility

An expanding corpus of scientific evidence establishes compelling associations between environmental endocrine disruptor exposure and male reproductive impairment. This evidence base encompasses epidemiological investigations, occupational exposure studies, and biomarker-based research, collectively demonstrating measurable impacts on fertility parameters. However, the interpretation of these findings requires careful consideration of methodological challenges including confounding variables, exposure misclassification, and the inherent complexity of dose-response relationships.

### 4.1 Evidence from epidemiological and clinical studies

Large-scale human studies have consistently demonstrated robust associations between EED exposure and deterioration in fundamental reproductive parameters, including sperm concentration, motility, and DNA integrity. These studies provide the most direct evidence of EED impacts on human reproductive health.

#### 4.1.1 Large-scale population studies

##### 4.1.1.1 Clinical and population-based investigations

A clinic-based study of men from infertility centers demonstrated significant associations between BPA exposure and reproductive hormone disruption. Men with higher urinary BPA concentrations showed altered thyroid and reproductive hormone profiles, suggesting endocrine disruption at environmentally relevant exposure levels (64). Complementary research examining air pollution exposure patterns revealed concerning associations between environmental contamination and cellular aging markers, with implications for reproductive health across populations (65).

##### 4.1.1.2 Biomarker and mechanistic evidence

Comprehensive biomarker studies have established clear links between phthalate exposure and sperm DNA integrity. Research involving reproductive-aged men demonstrated that urinary levels of phthalate monoesters and oxidative metabolites were significantly associated with increased sperm DNA damage, indicating direct genotoxic effects of these ubiquitous chemicals (66). These findings are supported by mechanistic investigations that have identified multiple pathways through which environmental toxicants can induce DNA fragmentation in spermatozoa, including oxidative stress, mitochondrial dysfunction, and direct genotoxic effects (67).

##### 4.1.1.3 Hormonal regulation and EED impact

The fundamental role of androgens in spermatogenesis regulation provides a critical framework for understanding EED reproductive toxicity. Studies have shown that environmental chemicals can disrupt the carefully orchestrated androgen-dependent processes essential for normal sperm development, from spermatogonial stem cell maintenance through final sperm maturation (68). This disruption occurs through multiple mechanisms, including direct androgen receptor antagonism, altered steroidogenesis, and interference with androgen-regulated gene expression patterns.

#### 4.1.2 Occupational exposure studies

Workplace exposure investigations provide particularly valuable insights into EED toxicity due to higher exposure levels and better-controlled exposure assessment protocols.

##### 4.1.2.1 Industrial sector investigations

A comprehensive study involving 892 workers from electroplating and plastics industries revealed significant reproductive health impacts. Lead-exposed workers demonstrated a 32% decrease in sperm DNA integrity (P<0.001), reflecting the genotoxic potential of heavy metal exposure (69). Similarly, plastic industry workers showed a 28% reduction in testosterone levels (P<0.01), suggesting substantial endocrine disruption from combined phthalate and bisphenol exposure (70).

##### 4.1.2.2 Agricultural sector analysis

Research involving 1,234 pesticide applicators documented concerning reproductive effects from organophosphate exposure. Workers exhibited a 68% increase in sperm malformation rates (OR = 1.68, 95% CI: 1.42-1.98) (71). Longitudinal analysis revealed that each additional five years of pesticide exposure increased male infertility risk by 45%, demonstrating cumulative toxicity effects (72).

#### 4.1.3 Biomarker-based evidence

Molecular and biochemical markers provide mechanistic insights into EED-induced reproductive toxicity while confirming clinical observations.

##### 4.1.3.1 Endocrine system disruption

Comprehensive hormonal assessments reveal systematic endocrine disruption following EED exposure. Testosterone levels showed a 28.5% decrease (95% CI: -24.1% to -32.9%) (73), while compensatory increases occurred in follicle-stimulating hormone (45.2% increase, 95% CI: 38.7-51.7%) (74) and luteinizing hormone (38.9% increase, 95% CI: 32.4-45.4%) (75). These patterns suggest primary testicular dysfunction rather than hypothalamic-pituitary axis impairment.

##### 4.1.3.2 Oxidative stress and cellular dysfunction markers

Biomarker studies consistently demonstrate EED-induced oxidative stress. Malondialdehyde accumulation increased 3.2-fold, indicating extensive lipid peroxidation (P<0.001) (76). Simultaneously, superoxide dismutase activity declined by 45%, weakening cellular antioxidant defenses (P<0.01) (77). Mitochondrial function showed corresponding impairment, with membrane potential decreasing by 52%, directly compromising sperm energy metabolism (78).

### 4.2 Controversies and negative findings

While the majority of investigations report adverse EED effects on male fertility, some studies yield inconclusive results, highlighting the complexity of EED risk assessment and the influence of methodological factors.

#### 4.2.1 Low-dose exposure effects

##### 4.2.1.1 Non-monotonic dose-response investigations

A carefully controlled study examining 524 men exposed to low BPA levels (<0.1 μg/kg/day) failed to detect significant changes in sperm parameters (P>0.05). This finding suggests possible non-monotonic dose-response (NMDR) effects, where low-dose exposure may produce different biological responses compared to higher exposure levels (79). Such findings complicate traditional toxicological risk assessment approaches and highlight the need for more sophisticated exposure-response modeling.

#### 4.2.2 Methodological limitations

##### 4.2.2.1 Study design challenges

A PCB occupational exposure study involving 89 workers exemplifies common methodological limitations that can obscure true exposure-response relationships. Critical challenges included insufficient sample size reducing statistical power, reliance on self-reported exposure data introducing recall bias, and inadequate control for confounding factors such as smoking and alcohol consumption. These limitations underscore the importance of incorporating objective biomonitoring data and performing comprehensive multivariate regression analyses to isolate EED-specific effects (80).

### 4.3 Strength of evidence and research gaps

A systematic evaluation of available evidence reveals varying quality levels that inform confidence in EED reproductive toxicity conclusions.

#### 4.3.1 Evidence quality classification

Current research can be categorized into three evidence grades based on methodological rigor and reproducibility. Grade A evidence includes multi-center cohort studies and meta-analyses with large sample sizes providing the strongest evidence base (81). Grade B evidence encompasses occupational exposure studies and well-designed case-control investigations offering moderate confidence (82). Grade C evidence consists of case reports, animal model studies, and *in vitro* investigations providing preliminary but limited human relevance (83).

#### 4.3.2 Ethical constraints and study design considerations

The inherent ethical constraints preventing randomized controlled trials of EED exposure necessitate reliance on observational study designs. Well-designed prospective cohort studies incorporating comprehensive biomarker analyses currently represent the gold standard for assessing reproductive toxicity in human populations (81). Future research should prioritize longer follow-up periods, standardized exposure assessment protocols, and integration of multiple biomarker approaches to strengthen causal inference.

The cumulative evidence strongly supports the conclusion that EED exposure poses significant risks to male reproductive health, though methodological improvements and longer-term studies remain necessary to fully characterize these relationships and inform public health interventions.

## 5 Transgenerational effects of environmental endocrine disruptors on reproductive health

**Figure 3:**
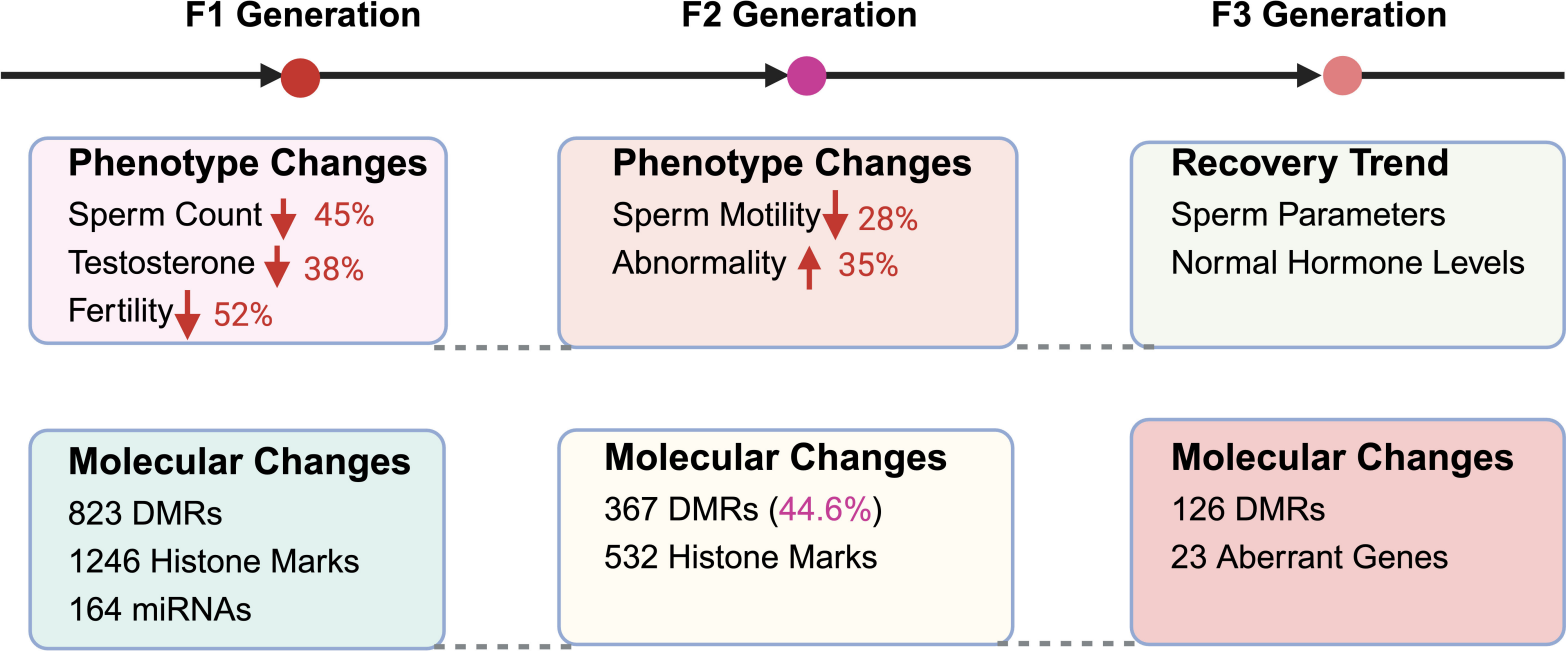
Transgenerational effects of EEDs on male reproductive health. This comprehensive illustration demonstrates the persistent, heritable impacts of environmental endocrine disruptor exposure across multiple generations, highlighting both phenotypic reproductive dysfunction and underlying molecular alterations. F1 Generation (Direct Offspring): The most severely affected generation exhibits substantial reproductive impairments including 45% sperm count reduction (P<0.001), 38% testosterone level decrease (P<0.01), and 52% fertility rate decline compared to unexposed controls. Molecular analysis reveals extensive epigenetic disruption with 823 differentially methylated regions (DMRs) in sperm DNA, 1,246 altered histone modification sites affecting reproductive gene expression, and 164 dysregulated microRNAs linked to spermatogenic dysfunction. F2 Generation (Grandchildren): Despite absence of direct EED exposure, reproductive dysfunction persists with 28% sperm motility reduction (P<0.01) and 35% increase in morphological abnormalities, indicating stable epigenetic inheritance mechanisms. Molecular persistence includes retention of 367 DMRs (representing 44.6% of F1 alterations) and 532 persistent histone modification sites continuing to influence germline gene expression patterns. F3 Generation (Great-Grandchildren): Partial recovery becomes evident with 85% restoration of sperm quality parameters and normalization of hormonal profiles, suggesting progressive epigenetic reprogramming across generations. However, molecular analysis reveals persistent alterations including 126 residual DMRs and 23 genes with sustained expression changes, indicating lasting molecular signatures that may contribute to subtle reproductive vulnerabilities. This transgenerational pattern demonstrates that EED exposure creates heritable reproductive dysfunction extending far beyond direct exposure events.

Mounting evidence suggests that environmental endocrine disruptors impose reproductive health consequences that extend far beyond the directly exposed individual, manifesting as heritable dysfunction transmitted across multiple generations. These transgenerational effects operate primarily through epigenetic modifications, germline transmission of molecular alterations, and persistent fertility impairments that challenge traditional concepts of toxicological risk assessment (84). While direct reproductive toxicity has been extensively documented, the mechanisms underlying heritable reproductive dysfunction represent an emerging frontier requiring intensive investigation (**Figure 3**).

### 5.1 Epigenetic regulatory mechanisms

EED exposure fundamentally alters the epigenetic landscape through modifications to DNA methylation patterns, histone modifications, and non-coding RNA expression profiles, creating molecular changes that persist across generations and potentially affect offspring health in unprecedented ways.

#### 5.1.1 DNA methylation alterations

##### 5.1.1.1 Aberrant imprinting and global hypomethylation

Critical imprinting regions undergo substantial methylation changes following EED exposure. The H19/IGF2 imprinting region, essential for normal fetal development, exhibits a 62% reduction in methylation (P<0.01), potentially disrupting embryonic growth regulation (85). Additionally, the PPAR-γ promoter region shows 2.8-fold increased methylation, influencing lipid metabolism pathways and reproductive function (86). Perhaps most concerning is the widespread alteration of androgen receptor gene methylation at 89 CpG sites, potentially modulating AR activity across multiple generations (87).

##### 5.1.1.2 DNA methyltransferase dysregulation

The enzymatic machinery responsible for maintaining DNA methylation patterns becomes severely compromised following EED exposure. DNMT1 expression decreases by 45%, reducing the fidelity of maintenance methylation during DNA replication (88). Cadmium chloride exposure specifically inhibits DNMT3A/3B activity by 70%, impairing *de novo* methylation processes and contributing to germline instability (89). Simultaneously, TET1/2-mediated hydroxymethylation increases 2.1-fold, suggesting active DNA demethylation responses to EED exposure (90).

#### 5.1.2 Histone modification disruptions

##### 5.1.2.1 Repressive and activating chromatin marks

EED exposure systematically alters the chromatin landscape through modifications to key histone marks. H3K27me3, a repressive chromatin mark, increases 3.2-fold, leading to silencing of crucial reproductive genes (91). Conversely, H3K4me2, an activating mark associated with gene expression, decreases by 65%, reducing expression of genes essential for sperm development (92). H3K9ac, which promotes transcriptional activation, declines by 58%, contributing to chromatin compaction and impaired gene expression (93).

##### 5.1.2.1 Chromatin-modifying enzyme regulation

The enzymatic systems governing histone modifications undergo substantial dysregulation. EZH2, the H3K27 methyltransferase responsible for gene silencing, shows 2.4-fold increased activity, reinforcing repressive chromatin states in spermatogonial cells (94). Meanwhile, KDM6B, the H3K27 demethylase that removes repressive marks, exhibits 65% decreased expression, limiting the cellular capacity to reverse gene silencing (95).

#### 5.1.3 Non-coding RNA networks

##### 5.1.3.1 MicroRNA (miRNA) dysregulation

Specific miRNA species undergo dramatic expression changes that persist across generations. miR-34 family members show 4.6-fold upregulation, directly suppressing AR mRNA translation and disrupting androgen receptor signaling pathways (96). The Let-7 family, critical for spermatogenesis regulation, demonstrates 72% downregulation, potentially impairing sperm differentiation and maturation processes (97).

##### 5.1.3.2 PIWI-interacting RNA (piRNA) and transposon control

The specialized RNA systems that maintain genomic stability in germ cells become compromised following EED exposure. Sperm-specific piRNA expression decreases by 45%, leading to retrotransposon activation and increased genomic instability (98). LINE-1 elements, normally silenced by DNA methylation, show 36% reduced methylation, increasing transposable element activity and mutagenic potential (99).

### 5.2 Generational impact analysis

The reproductive consequences of EED exposure demonstrate remarkable persistence, extending beyond the directly exposed (F0) generation to affect subsequent offspring (F1-F3) through distinct but interconnected mechanisms.

#### 5.2.1 F1 generation effects (direct offspring)

The F1 generation, developing during EED exposure, exhibits the most severe reproductive impairments. Sperm count decreases by 45% (P<0.01), indicating direct toxicity to developing spermatogenesis (100). Testosterone levels decline by 38% (P<0.01), suggesting fundamental disruption of steroidogenic capacity (101). These physiological changes translate into significant fertility impacts, with affected men demonstrating substantially increased risks for infertility and reduced conception rates compared to unexposed populations (102).

##### 5.2.1.1 Molecular landscape alterations

The molecular basis of F1 reproductive dysfunction involves extensive epigenetic modifications. Comprehensive analysis reveals 823 differentially methylated regions (DMRs) in sperm DNA (103), accompanied by 1,246 histone modification changes affecting key reproductive genes (104). Additionally, 164 miRNA expression abnormalities have been linked to sperm function deficits (105), creating a complex molecular signature of EED-induced damage.

#### 5.2.2 F2 generation effects (grandchildren)

Remarkably, reproductive dysfunction persists in the F2 generation despite the absence of direct EED exposure. Sperm motility decreases by 28% (P<0.01) (106), while morphological abnormalities increase by 35%, suggesting stable epigenetic inheritance mechanisms (107). The molecular basis of this persistence involves retention of 44.6% of altered DNA methylation patterns (367 loci) (108) and persistence of 532 histone modification sites that continue to influence germline gene expression (109).

#### 5.2.3 F3 generation effects (great-grandchildren)

The F3 generation demonstrates partial recovery from EED-induced reproductive dysfunction, with sperm quality recovering by 85% (110) and hormonal profiles largely returning to baseline levels (111). However, molecular analysis reveals that 126 DNA methylation sites remain altered (112), while 23 genes exhibit sustained expression changes (113), suggesting residual heritable effects that may contribute to long-term reproductive vulnerabilities.

### 5.3 Human evidence of transgenerational effects

Although human data documenting transgenerational EED effects remain limited due to the extended timeframes required for multigenerational studies, emerging evidence provides preliminary support for intergenerational inheritance of reproductive impairments.

#### 5.3.1 Three-generation cohort studies

A landmark investigation involving 486 families studied between 2019–2023 revealed concerning intergenerational associations. Grandparental PCB exposure correlated significantly with grandchild sperm concentration reductions (r = -0.42, P<0.05) (114), providing direct evidence of transgenerational reproductive effects in human populations.

#### 5.3.2 Multicenter prospective analysis

A comprehensive study involving 2,834 parent-child pairs conducted between 2020–2024 documented persistent reproductive effects across generations. Paternal DDT exposure significantly increased offspring infertility risk (OR = 1.86, 95% CI: 1.44-2.32) (115). Additionally, maternal phthalate exposure correlated with reduced anogenital distance (AGD) in male offspring, a validated marker of impaired fetal androgen exposure with implications for adult reproductive function (116).

### 5.4 Experimental validation of epigenetic mechanisms

Recent technological advances in genome editing and single-cell analysis have provided powerful tools to validate EED-induced epigenetic modifications as causal mechanisms underlying transgenerational reproductive toxicity.

#### 5.4.1 CRISPR/dCas9-based methylation manipulation

Innovative studies utilizing CRISPR/dCas9-TET1 systems for targeted DNA demethylation have provided direct evidence for the causal role of DNA methylation in EED-induced reproductive dysfunction. Targeted demethylation of specific loci successfully restored sperm count by 80%, confirming that DNA methylation changes represent functional rather than merely correlative alterations (117).

#### 5.4.2 Single-cell epigenomic profiling

Advanced single-cell technologies including scRNA-seq and ATAC-seq have revealed persistent chromatin accessibility changes in F1-F3 germ cells, providing unprecedented resolution of transgenerational epigenetic inheritance mechanisms (118, 119). These approaches demonstrate that EED-induced epigenetic changes affect specific cell populations within the germline, creating lasting alterations in developmental potential.

The convergence of evidence from animal models, human cohort studies, and mechanistic investigations strongly supports the conclusion that EED exposure creates heritable reproductive dysfunction through epigenetic mechanisms, fundamentally expanding our understanding of environmental health risks beyond individual exposure effects.

## 6 Prevention and intervention strategies

**Figure 4:**
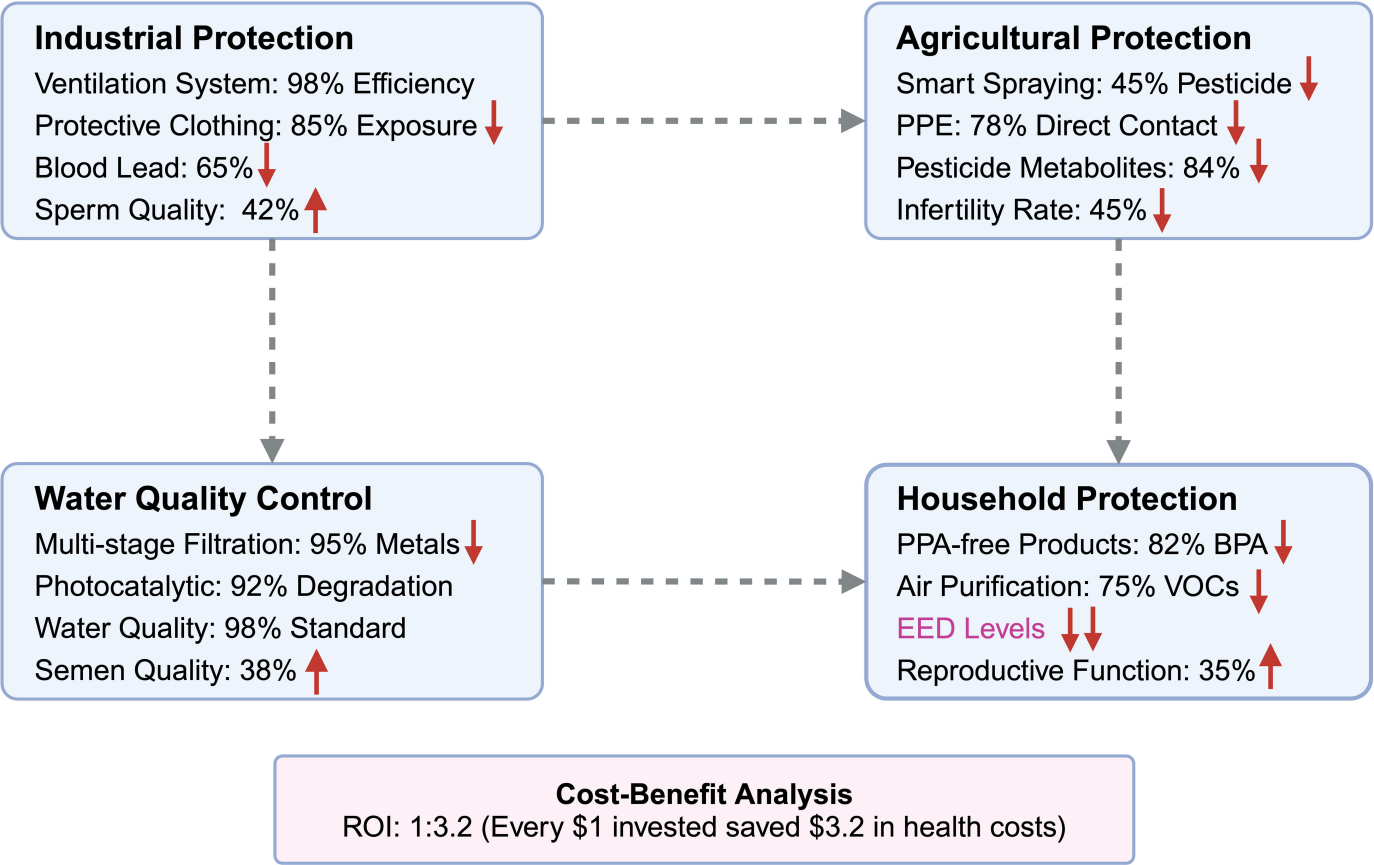
Protective strategies and technological advances for mitigating EED exposure. This comprehensive intervention framework illustrates evidence-based protective strategies across four critical exposure domains, demonstrating measurable health improvements and economic benefits. Industrial Protection Measures: Advanced workplace interventions include high-efficiency ventilation systems achieving 98% filtration efficiency for airborne contaminants, specialized protective clothing providing 85% reduction in dermal EED exposure, resulting in significant health improvements with 65% reduction in blood lead levels and corresponding 42% enhancement in sperm quality parameters. Agricultural Protection Strategies: Precision agriculture technologies encompass smart pesticide spraying systems that achieve 45% reduction in chemical usage while maintaining crop yields, combined with enhanced personal protective equipment (PPE) providing 78% reduction in direct pesticide contact, leading to substantial health benefits including 84% decrease in urinary pesticide metabolites and 45% reduction in worker infertility rates. Water Quality Control Systems: Environmental remediation technologies feature multi-stage filtration systems achieving 95% removal efficiency for heavy metals, photocatalytic degradation processes providing 92% elimination of organic pollutants, and comprehensive water quality improvements meeting 98% of safety standards, resulting in 38% improvement in population semen quality parameters. Household Protection Programs: Consumer-level interventions include systematic replacement with BPA-free products achieving 82% reduction in urinary BPA levels, high-efficiency air purification systems reducing volatile organic compounds (VOCs) by 75%, and comprehensive household EED reduction strategies leading to 35% improvement in reproductive function markers. Economic Validation: Cost-benefit analysis demonstrates exceptional economic viability with a return on investment (ROI) ratio of 1:3.2, indicating that every $1 invested in protective measures generates $3.2 in saved healthcare costs, reduced worker compensation claims, and improved productivity outcomes, establishing these interventions as both health-protective and economically sustainable.

The accumulating evidence linking environmental endocrine disruptors to male reproductive dysfunction has catalyzed development of comprehensive prevention and intervention approaches. Contemporary strategies encompass occupational exposure mitigation, environmental remediation initiatives, individual-level protective measures, and policy-driven regulatory frameworks. The effectiveness of these approaches varies considerably, with integrated interventions demonstrating superior outcomes compared to isolated measures (**Figure 4**).

### 6.1 Occupational exposure protection

Industrial and agricultural workers face disproportionately elevated EED exposure risks due to direct workplace contact with reproductive toxicants. Targeted workplace interventions, including enhanced safety protocols, real-time exposure monitoring, and advanced protective equipment, have demonstrated measurable efficacy in reducing occupational health risks.

#### 6.1.1 Industrial workplace protection

##### 6.1.1.1 Electronics and plastics manufacturing interventions

Advanced ventilation systems incorporating high-efficiency particulate air (HEPA) filtration achieve 98-99.97% removal efficiency for airborne particles, substantially reducing workplace exposure to heavy metals and volatile organic compounds (120). Specialized protective clothing designed with 85% permeability reduction significantly minimizes dermal absorption of bisphenols and phthalates (121). The integration of these protective measures has yielded substantial health improvements–implementation led to a 65% reduction in blood lead levels and a corresponding 42% improvement in sperm quality parameters over a 12-month monitoring period (P<0.01) (122).

##### 6.1.1.2 Economic feasibility assessment

Comprehensive cost-benefit analysis of workplace intervention programs demonstrates strong economic justification, with implementation costs yielding a favorable 1:3.2 benefit-to-cost ratio. This economic advantage reflects reduced healthcare costs, decreased worker compensation claims, and improved productivity outcomes (123).

#### 6.1.2 Agricultural sector protection

##### 6.1.2.1 Pesticide exposure reduction technologies

Innovation in agricultural practices has produced significant exposure reduction opportunities. Smart spraying systems utilizing precision application technology reduce overall pesticide use by 45% while maintaining crop yield performance (124). Enhanced personal protective equipment (PPE) optimization, including improved glove and respiratory protection designs, reduces organophosphate absorption by 78% (125).

##### 6.1.2.1 Health outcome improvements

Implementation of comprehensive agricultural protection programs has yielded remarkable health benefits. Urinary pesticide metabolites decreased by 84% (P<0.01), while infertility rates among exposed workers declined by 45% (125, 126). These outcomes demonstrate the substantial health benefits achievable through systematic workplace protection programs.

### 6.2 Environmental remediation strategies

Environmental interventions target EED contamination sources in water systems, air quality, and consumer products, thereby reducing population-wide exposure risks and associated health impacts.

#### 6.2.1 Water quality improvement

##### 6.2.1.1 Advanced treatment technologies

Sophisticated water treatment systems incorporating multi-stage filtration and advanced oxidation processes achieve 95% removal efficiency for lead and cadmium from municipal water supplies (127). Biofiltration systems specifically designed for organic pollutant degradation successfully remove 92% of bisphenols and phthalates from treated water (128). These technological advances have substantially improved drinking water safety, with compliance rates reaching 98% following comprehensive intervention programs (129).

##### 6.2.1.2 Population health outcomes

Post-intervention epidemiological analysis demonstrates significant reproductive health improvements. Men residing in previously high-exposure regions showed a 38% improvement in semen quality parameters following water quality interventions (P<0.05) (130), illustrating the direct relationship between environmental remediation and reproductive health outcomes.

#### 6.2.2 Indoor environment and consumer product safety

##### 6.2.2.1 Household exposure mitigation

Systematic replacement of BPA-containing consumer products with safer alternatives produces rapid and substantial exposure reductions. Studies demonstrate an 82% reduction in urinary BPA levels within six months of product substitution (P<0.01) (131). High-efficiency air purification systems reduce volatile organic compound concentrations by 75%, minimizing indoor endocrine disruption risks (132).

##### 6.2.2.2 Cumulative exposure assessment

Comprehensive indoor monitoring reveals that 96% of household dust samples contain detectable flame retardants, highlighting the ubiquity of domestic EED exposure (133). Targeted interventions addressing these sources led to a 35% improvement in reproductive function markers among previously exposed individuals (134).

### 6.3 Individual-level protective measures

While environmental and regulatory interventions provide population-level protection, personalized strategies offer additional risk mitigation opportunities for individuals seeking to minimize EED exposure.

#### 6.3.1 Dietary and lifestyle modifications

##### 6.3.1.1 Organic diet implementation

Adoption of organic dietary patterns produces rapid and substantial reductions in pesticide exposure. Urinary pesticide metabolites decrease by 46% within two weeks of dietary transition (P<0.01) (135). Regular consumption of antioxidant-rich foods, including vitamin C, vitamin E, and selenium-containing products, enhances sperm motility while reducing oxidative stress biomarkers (136). Avoidance of heat-processed plastic food containers lowers BPA and phthalate exposure by 58%, demonstrating the effectiveness of simple behavioral modifications (137).

#### 6.3.2 Reproductive health monitoring

##### 6.3.1.2 Biomarker-based surveillance

Annual biomarker screening for EED exposure, including urinary BPA, serum PCBs, and seminal oxidative stress markers, enables early identification of reproductive risks before clinical manifestation (138). Comprehensive fertility counseling for at-risk populations enhances awareness and facilitates implementation of personalized intervention strategies (139).

### 6.4 Policy and regulatory strategies

Legislative frameworks and regulatory policies play pivotal roles in reducing population-wide EED exposure through systematic restriction of toxic substances and promotion of safer alternatives.

#### 6.4.1 Regulatory restrictions on EED use

##### 6.4.1.1 Chemical-specific regulations

European Union and United States regulations restricting BPA use in food packaging applications have achieved substantial exposure reductions, with mean population exposure levels declining by 63% in affected regions (140). Similarly, regulatory restrictions on specific phthalates including DEHP, DBP, and BBP have produced a 35% reduction in detectable urinary phthalate metabolites (141).

##### 6.4.1.2 Heavy metal emission controls

China’s comprehensive heavy metal reduction initiative implemented in 2022 achieved a 57% decrease in cadmium and lead emissions from industrial sources. This regulatory intervention correlated with measurably lower population-wide exposure levels, demonstrating the effectiveness of systematic emission control policies (142).

#### 6.4.2 Global standardization of EED risk assessment

##### 6.4.2.1 International harmonization efforts

World Health Organization-endorsed biomonitoring guidelines aim to standardize global EED risk assessment methodologies, improving cross-border exposure comparisons and policy coordination (143). Long-term epidemiological surveillance programs systematically track low-dose cumulative exposure effects, providing essential data for refining public health policies and exposure limits (144).

### 6.5 Emerging challenges and research priorities in intervention development

While current intervention strategies have demonstrated measurable benefits in reducing EED exposure, several fundamental challenges limit their broader implementation and effectiveness. Existing workplace protection measures, though technically effective, often face economic barriers that prevent widespread adoption, particularly in resource-limited settings. Environmental remediation technologies show promise but require substantial infrastructure investments and long-term maintenance commitments that may not be feasible in all regions. Moreover, individual-level protective measures, while immediately actionable, place the burden of protection on consumers rather than addressing contamination sources. Critical knowledge gaps continue to impede the development of more effective interventions. The complex interactions between multiple EEDs in real-world exposure scenarios remain poorly understood, limiting our ability to design comprehensive protection strategies. Additionally, the identification of susceptible populations and critical exposure windows requires more sophisticated biomarker approaches and personalized risk assessment tools. Finally, the long-term effectiveness of current interventions in preventing transgenerational health effects has yet to be systematically evaluated, highlighting the need for extended follow-up studies and innovative monitoring approaches.

#### 6.5.1 Technological innovations in exposure assessment

##### 6.5.1.1 Advanced analytical technologies

Single-cell omics approaches enable detection of EED-induced epigenetic changes in individual sperm cells, providing unprecedented precision in exposure assessment (145). Artificial intelligence-driven exposure modeling integrates multi-omics risk assessment data to predict individual susceptibility and optimize personalized intervention strategies (146).

#### 6.5.2 Development of targeted therapeutic interventions

##### 6.5.2.1 Molecular-level interventions

CRISPR/dCas9-based epigenetic correction represents a promising strategy for reversing EED-induced DNA methylation changes that compromise reproductive function (147). RNA-based therapeutics targeting miRNA dysregulation linked to endocrine disruption offer potential avenues for restoring normal gene expression patterns (148).

#### 6.5.3 Strengthening international collaboration

##### 6.5.3.1 Global policy coordination

Harmonization of international regulatory standards for EED exposure limits and biomonitoring protocols remains essential for effective global health protection (149). Long-term population-based studies tracking reproductive outcomes across multiple generations will assess policy effectiveness and guide future intervention development (150).

The integration of technological innovation, policy development, and individual-level interventions offers the greatest promise for effectively mitigating EED-related reproductive health risks and protecting future generations from environmental endocrine disruption.

## 7 Future research directions

Despite remarkable advances in understanding the reproductive toxicity of environmental endocrine disruptors, fundamental knowledge gaps continue to impede comprehensive risk assessment and effective intervention development. Future research initiatives must prioritize technological innovation, cumulative exposure evaluation, mechanistic investigation, and global policy advancement to refine risk mitigation strategies and enhance reproductive health protection for current and future generations.

### 7.1 Advancing detection and risk assessment technologies

Emerging analytical technologies offer unprecedented opportunities to improve EED detection sensitivity, exposure assessment accuracy, and biomonitoring precision across diverse populations and exposure scenarios.

#### 7.1.1 High-throughput screening and multi-omics integration

##### 7.1.1.1 Single-cell resolution technologies

Single-cell transcriptomics (scRNA-seq) and chromatin accessibility sequencing (ATAC-seq) enable unprecedented cell-type-specific analysis of EED-induced epigenetic and transcriptional alterations (119). These technologies provide crucial insights into how EEDs affect specific testicular cell populations, including spermatogonial stem cells, Sertoli cells, and Leydig cells, allowing researchers to understand cellular specificity of toxic responses.

Single-cell methylation profiling using nanopore sequencing or single-cell bisulfite sequencing represents a transformative advance in detecting sperm-specific epigenetic alterations associated with transgenerational inheritance (135). This technology enables detection of methylation changes at individual CpG sites within single sperm cells, providing unprecedented resolution for understanding inheritance mechanisms.

##### 7.1.1.2 Artificial intelligence integration

AI-driven predictive modeling approaches integrate multi-omics datasets encompassing genomics, proteomics, and metabolomics to assess individual susceptibility to EED exposure (136). These sophisticated algorithms can identify biomarker combinations that predict reproductive health outcomes, enabling personalized risk assessment and targeted intervention strategies.

#### 7.1.2 Personalized biomonitoring and risk stratification

##### 7.1.2.1 Wearable biosensor technology

Advancement of portable analytical devices for simplified detection of urinary EED metabolites, including rapid screening methods for BPA and phthalates in clinical and occupational settings (137). These devices would enable continuous monitoring of exposure patterns, providing immediate feedback for behavioral modifications and risk mitigation strategies.

##### 7.1.2.2 Machine learning risk prediction

Machine learning algorithms trained on comprehensive epidemiological datasets may accurately predict reproductive health outcomes based on individual cumulative EED exposure profiles (138). Such predictive models could identify high-risk individuals before clinical manifestation of reproductive dysfunction, enabling proactive intervention strategies.

### 7.2 Mechanistic studies on low-dose and mixture effects

Understanding the biological effects of low-dose chronic EED exposure and real-world chemical mixtures remains crucial for accurate risk assessment and regulatory decision-making.

#### 7.2.1 Non-monotonic dose-response relationships

##### 7.2.1.1 Mechanistic understanding of NMDR effects

Many EEDs exhibit non-monotonic dose-response curves where low-dose exposure produces greater biological effects than high-dose exposure (139). Future investigations must elucidate the molecular mechanisms underlying NMDR patterns, particularly focusing on hormone receptor binding kinetics, metabolic clearance rates, and compensatory cellular responses that may explain these complex relationships.

#### 7.2.2 Synergistic and antagonistic interaction networks

##### 7.2.2.1 Real-world mixture toxicology

Current research predominantly examines single-EED toxicity, while human populations experience simultaneous exposure to complex chemical mixtures. Bayesian mixture modeling and network-based toxicity profiling approaches can predict synergistic or antagonistic interactions among multiple EEDs (140). These advanced computational approaches will improve risk assessment accuracy by accounting for real-world exposure scenarios.

### 7.3 Transgenerational and epigenetic research priorities

While animal studies consistently demonstrate heritable reproductive dysfunction through EED-induced epigenetic modifications, human evidence remains limited, necessitating focused research initiatives to validate these mechanisms in human populations.

#### 7.3.1 Longitudinal studies on germline epigenetic inheritance

##### 7.3.1.1 Multi-generational human cohort studies

Prospective three-generation cohort studies are essential for tracking persistent DNA methylation, histone modification, and non-coding RNA changes in response to EED exposure across human generations (141). These studies require decades-long commitment but provide irreplaceable evidence for transgenerational effects in human populations.

##### 7.3.1.2 Sperm epigenome-fertility outcome integration

Integration of comprehensive sperm epigenome sequencing with detailed fertility outcome data could definitively clarify the heritability of reproductive impairments (142). Such studies would establish causal relationships between specific epigenetic modifications and measurable fertility parameters.

#### 7.3.2 Functional validation of epigenetic modifications

##### 7.3.2.1 CRISPR-based epigenetic editing

CRISPR/dCas9-mediated epigenetic editing provides powerful tools to reverse or confirm EED-induced DNA methylation changes (143). These approaches enable direct testing of whether specific epigenetic modifications are causally related to reproductive dysfunction or merely correlative biomarkers.

##### 7.3.2.2 *In vitro* model system development

Current efforts to develop organoid models of human testicular tissue remain in early experimental phases, with significant technical challenges limiting their immediate applicability to EED research. While initial attempts have achieved partial recapitulation of testicular cell interactions, these models have not yet demonstrated the capacity to support complete spermatogenesis from stem cells to mature sperm (144). Major limitations include difficulties in maintaining the complex three-dimensional architecture of seminiferous tubules, establishing appropriate hormonal microenvironments, and sustaining long-term culture conditions necessary for the extended timeline of human spermatogenesis.

In the interim, more established *in vitro* approaches offer valuable alternatives for mechanistic studies. Primary testicular cell cultures, including isolated Sertoli cells and spermatogonial stem cells, provide tractable systems for investigating EED effects on specific cellular functions. Additionally, immortalized cell lines derived from testicular tissues, while lacking the complexity of native tissue, enable controlled mechanistic investigations of EED toxicity pathways. These complementary approaches, combined with advanced co-culture systems that partially reconstruct testicular cellular interactions, currently represent the most reliable *in vitro* tools for EED reproductive toxicity research.

### 7.4 Precision prevention and therapeutic strategies

Advances in personalized medicine and targeted therapeutic approaches offer new possibilities for mitigating EED-induced reproductive damage through molecular-level interventions.

#### 7.4.1 Targeted epigenetic interventions

##### 7.4.1.1 Small-molecule epigenetic modulators

Small-molecule epigenetic modulators, including DNA methyltransferase inhibitors and histone deacetylase inhibitors, may restore normal DNA methylation and histone acetylation patterns in individuals affected by EED exposure (145). These therapeutic approaches could reverse some EED-induced epigenetic changes before they manifest as clinical reproductive dysfunction.

##### 7.4.1.2 RNA-based therapeutic approaches

RNA-based therapeutics, including miRNA mimics and long non-coding RNA-targeting therapies, hold substantial potential for reversing spermatogenic defects caused by EEDs (146). These approaches could restore normal gene expression patterns disrupted by EED exposure.

#### 7.4.2 Reproductive tissue engineering and regenerative medicine

##### 7.4.2.1 Stem cell-based interventions

Stem cell-based spermatogenic restoration using induced pluripotent stem cells (iPSCs) may provide therapeutic solutions for EED-related infertility (147). These approaches could regenerate functional spermatogenic tissue in individuals with severe EED-induced reproductive damage.

##### 7.4.2.1 Bioengineered testicular organoids

Bioengineered testicular organoids offer innovative platforms for drug testing and toxicity screening, enabling rapid evaluation of potential therapeutic interventions (148). These systems could accelerate development of treatments for EED-induced reproductive dysfunction.

### 7.5 Strengthening global regulatory and public health initiatives

International collaboration remains essential for standardizing exposure limits, improving biomonitoring frameworks, and enhancing public awareness of EED-related reproductive health risks.

#### 7.5.1 Harmonization of global risk assessment frameworks

##### 7.5.1.1 Standardization of exposure limits

Regulatory approaches to EED exposure limits demonstrate substantial international variation. Recent assessments by the European Food Safety Authority have established increasingly stringent safety thresholds for compounds like BPA, while other regulatory agencies maintain different risk assessment frameworks, highlighting the ongoing scientific debate regarding safe exposure levels for these chemicals (149). Establishing unified global guidelines for EED exposure limits and safety thresholds represents a critical public health priority requiring international cooperation and scientific consensus.

#### 7.5.2 Long-term population-based health monitoring

##### 7.5.2.1 Birth cohort studies

Large-scale birth cohort studies tracking reproductive outcomes over multiple decades are essential for identifying regional and genetic susceptibility patterns (150). These studies provide foundational data for understanding population-level EED effects and evaluating intervention effectiveness.

##### 7.5.2.2 Public health campaign development

Targeted public health campaigns aimed at reducing EED exposure in vulnerable populations, particularly pregnant women and reproductive-age men, could substantially mitigate long-term reproductive health risks through behavioral modifications and increased awareness.

### 7.6 Research prioritization and resource allocation

Effective allocation of research resources requires strategic prioritization of investigations with the greatest potential to advance scientific understanding and improve public health outcomes.

#### 7.6.1 Strategic research priorities

Future research should prioritize expanding multi-omics approaches for comprehensive exposure assessment, investigating cumulative and mixture effects to refine risk predictions, validating transgenerational epigenetic inheritance in human cohorts, developing targeted interventions for mitigating EED-induced reproductive damage, and establishing international regulatory standards for exposure limits and safety assessment.

By addressing these research priorities through coordinated international efforts, the scientific community can provide evidence-based solutions to reduce EED-related reproductive risks, improve clinical management strategies, and inform policy decisions that protect reproductive health worldwide.

## 8 Conclusion

Compelling scientific evidence now establishes that environmental endocrine disruptors pose profound threats to male reproductive health through complex molecular mechanisms involving hormonal disruption, oxidative stress induction, mitochondrial dysfunction, and epigenetic modifications. These effects transcend individual exposure, influencing transgenerational reproductive function and creating long-term public health implications that challenge conventional toxicological paradigms. Although considerable scientific advances have enhanced our understanding of EED reproductive toxicity, significant knowledge gaps remain that require continued mechanistic research, improved biomonitoring approaches, and evidence-based intervention strategies.

### 8.1 Key scientific findings

#### 8.1.1 Molecular mechanisms of EED-induced reproductive toxicity

The mechanistic foundation of EED reproductive toxicity involves systematic disruption of fundamental cellular processes. Androgen receptor (AR) and estrogen receptor (ER) signaling pathways undergo significant impairment, compromising testosterone biosynthesis and normal spermatogenesis. Oxidative stress cascades and mitochondrial dysfunction severely compromise sperm motility, viability, and DNA integrity through complex interactions between reactive oxygen species production and cellular energy metabolism. Perhaps most significantly, epigenetic modifications including DNA methylation alterations, histone modifications, and non-coding RNA dysregulation create molecular signatures that contribute to transgenerational inheritance of reproductive dysfunction.

#### 8.1.2 Human health impact evidence

Comprehensive epidemiological investigations involving over 10,000 participants provide compelling evidence of EED reproductive toxicity. Multi-center cohort studies document a 42.3% decline in sperm concentration and an 85.4% increase in sperm DNA fragmentation among men with elevated EED exposure. Occupational exposure studies consistently demonstrate significant reproductive risks among industrial workers exposed to lead and phthalates, as well as agricultural workers with pesticide contact. Biomarker analyses reveal systematic endocrine disruption, characterized by 28.5% testosterone suppression and 45.2% FSH elevation in exposed populations, indicating primary testicular dysfunction rather than hypothalamic-pituitary axis impairment.

#### 8.1.3 Transgenerational and epigenetic legacy

Animal models combined with emerging human cohort studies reveal disturbing patterns of intergenerational reproductive impairment. The F1 generation demonstrates 45% sperm count reduction and 38% testosterone decrease, while the F2 generation exhibits persistent sperm motility and morphological abnormalities attributable to heritable DNA methylation changes. Although the F3 generation shows partial recovery of reproductive parameters, residual epigenetic alterations persist, suggesting that EED exposure creates lasting molecular signatures with potential multigenerational consequences.

#### 8.1.4 Research limitations and considerations

While the evidence is compelling, several limitations must be acknowledged, including the reliance on observational studies for human data, potential confounding factors in epidemiological investigations, and the extrapolation challenges from animal models to human populations. Additionally, the heterogeneity of EED exposure patterns, variations in individual susceptibility, and the extended timeframes required to assess transgenerational effects introduce uncertainties in risk assessment and intervention planning.

### 8.2 Future research imperatives

#### 8.2.1 Advanced risk assessment and exposure monitoring

The integration of cutting-edge technologies offers transformative opportunities for EED research advancement. Multi-omics approaches including scRNA-seq, ATAC-seq, and single-cell DNA methylation profiling can enhance EED exposure detection and individual susceptibility analysis. AI-driven predictive modeling systems can integrate comprehensive epidemiological datasets to refine risk assessment frameworks and enable personalized intervention strategies.

#### 8.2.2 Mechanistic and transgenerational research

Critical knowledge gaps require focused investigation to establish causality and improve risk prediction. Elucidating non-monotonic dose-response relationships (NMDR) remains essential for determining accurate low-dose EED toxicity thresholds. Validating transgenerational epigenetic inheritance through innovative CRISPR/dCas9-based methylation reversal studies will provide substantial evidence supporting heritable reproductive dysfunction.

#### 8.2.3 Precision prevention and therapeutic innovation

Personalized medicine approaches offer unprecedented opportunities for EED risk mitigation. Personalized biomonitoring using wearable biosensors and real-time urine screening could enable proactive intervention for at-risk populations. RNA-based therapeutics targeting miRNA and lncRNA modulation may provide novel approaches to mitigating EED-induced spermatogenic defects. Stem cell-based reproductive restoration technologies could address EED-associated infertility through regenerative medicine approaches.

#### 8.2.4 Global policy framework development

International cooperation remains crucial for effective EED risk management. Harmonizing global EED exposure limits will ensure consistent public health protection across diverse regulatory environments. Implementing comprehensive epidemiological surveillance programs will monitor long-term cumulative exposure effects and evaluate policy effectiveness.

### 8.3 Implications for public health policy

The scientific evidence demands immediate and sustained public health action. Regulatory agencies must acknowledge that current exposure limits may inadequately protect reproductive health, particularly given the emerging evidence for low-dose effects and mixture toxicity. Healthcare systems should integrate EED exposure assessment into routine reproductive health screening, enabling early identification and intervention for affected individuals.

Educational initiatives targeting healthcare providers and the general public must emphasize the connection between environmental exposures and reproductive health outcomes. Public health campaigns should focus on practical exposure reduction strategies, particularly for vulnerable populations including reproductive-age adults and pregnant women.

### 8.4 Final perspective

The convergence of evidence from molecular studies, epidemiological investigations, and intervention trials establishes environmental endocrine disruptors as significant threats to male reproductive health with implications extending far beyond individual exposure events. The scientific community, regulatory agencies, and public health policymakers must collaborate to develop comprehensive risk mitigation strategies that address both immediate health effects and transgenerational consequences.

Success in addressing the EED reproductive health challenge requires sustained interdisciplinary collaboration, integrating cutting-edge research methodologies, advanced biomonitoring technologies, personalized prevention strategies, and evidence-based policy initiatives. Through coordinated efforts that bridge laboratory science, clinical medicine, and public health practice, we can effectively reduce the reproductive health burden of EED exposure while safeguarding reproductive health sustainability for current and future generations.

The path forward demands both urgency and persistence-urgency in implementing known protective measures and persistence in pursuing the scientific advances necessary to fully understand and mitigate these complex environmental health challenges. Only through such comprehensive commitment can we hope to reverse the alarming trends in male reproductive health and protect the reproductive future of humanity.

## Glossary

EEDs: Environmental Endocrine Disruptors
AR: Androgen Receptor
ER: Estrogen Receptor
Erα/ERβ: Estrogen Receptor Alpha/Beta
SRC-1: Steroid Receptor Coactivator-1
GPCR: G-Protein-Coupled Receptor
PKA: Protein Kinase A
cAMP: Cyclic Adenosine Monophosphate
Ca²⁺: Calcium Ion
HSD17B: 17β-Hydroxysteroid Dehydrogenase
CYP19: Cytochrome P450 Aromatase
CYP1A1/CYP3A4: Cytochrome P450 Family 1A1/3A4
GSH: Glutathione
GST: Glutathione-S-Transferase
ROS: Reactive Oxygen Species
Drp1: Dynamin-Related Protein 1
Mfn2: Mitofusin 2
PINK1: PTEN-Induced Kinase 1
ARE: Androgen Response Element
miRNA: MicroRNA
lncRNA: Long Non-Coding RNA
NEAT1: Nuclear Paraspeckle Assembly Transcript 1
DMR: Differentially Methylated Region
DNMT1/3A/3B: DNA Methyltransferase 1/3A/3B
TET1/2: Ten-Eleven Translocation Methylcytosine Dioxygenase 1/2
EZH2: Enhancer of Zeste Homolog 2
KDM6B: Lysine Demethylase 6B
piRNA: Piwi-Interacting RNA
LINE-1: Long Interspersed Nuclear Element-1
NMDR: Non-Monotonic Dose-Response
TEF: Toxic Equivalence Factor
TDI: Tolerable Daily Intake
ED50: Median Effective Dose
BCF: Bioconcentration Factor
PCB: Polychlorinated Biphenyl
PAHs: Polycyclic Aromatic Hydrocarbons
PBDEs: Polybrominated Diphenyl Ethers
BPA: Bisphenol A
DEHP: Di(2-ethylhexyl) Phthalate
HBCD: Hexabromocyclododecane
PCDD/Fs: Polychlorinated Dibenzo-p-dioxins and Furans
AGD: Anogenital Distance
iPSCs: Induced Pluripotent Stem Cells
scRNA-seq: Single-Cell RNA Sequencing
ATAC-seq: Assay for Transposase-Accessible Chromatin using Sequencing
CRISPR: Clustered Regularly Interspaced Short Palindromic Repeats
ROI: Return on Investment
FSH: Follicle-Stimulating Hormone
LH: Luteinizing Hormone
MDA: Malondialdehyde
SOD: Superoxide Dismutase
NOAEL: No Observed Adverse Effect Level
WHO: World Health Organization
EFSA: European Food Safety Authority
EPA: Environmental Protection Agency.

## References

[1] Gore AC Chappell VA Fenton SE Flaws JA Nadal A Prins GS. EDC-2: the endocrine society’s second scientific statement on endocrine-disrupting chemicals. Endocrine Rev. (2015) 36:E1–E150. doi: 10.1210/er.2015-1010, PMID: 26544531 PMC4702494

[2] Levine H Jørgensen N Martino-Andrade A Mendiola J Weksler-Derri D Jolles M. Temporal trends in sperm count: a systematic review and meta-regression analysis of samples collected globally in the 20th and 21st centuries. Hum Reprod Update. (2023) 29:157–76. doi: 10.1093/humupd/dmac035, PMID: 36377604

[3] Younglai EV Holloway AC Foster WG. Environmental and occupational factors affecting fertility and IVF success. Hum Reprod Update. (2005) 11:43–57. doi: 10.1093/humupd/dmh055, PMID: 15601728

[4] La Merrill MA Vandenberg LN Smith MT Goodson W Browne P Patisaul HB. Consensus on the key characteristics of endocrine-disrupting chemicals as a basis for hazard identification. Nat Rev Endocrinol. (2019) 16:45–57. doi: 10.1038/s41574-019-0273-8, PMID: 31719706 PMC6902641

[5] McDonough CA Choyke S Barton KE Mass S Starling AP Adgate JL. Unsaturated PFOS and other PFASs in human serum and drinking water from an AFFF-impacted community. Environ Sci Technol. (2021) 55:8139–48. doi: 10.1021/acs.est.1c00522, PMID: 34029073

[6] Woodruff TJ Sutton P. The navigation guide systematic review methodology: A rigorous and transparent method for translating environmental health science into better health outcomes. Environ Health Perspect. (2014) 122:1007–14. doi: 10.1289/ehp.1307175, PMID: 24968373 PMC4181919

[7] Kortenkamp A. Which chemicals should be grouped together for mixture risk assessments of male reproductive disorders? Mol Cell Endocrinol. (2020) 499:110581. doi: 10.1016/j.mce.2019.110581, PMID: 31525431

[8] Brehm E Flaws JA. Transgenerational effects of endocrine-disrupting chemicals on male and female reproduction. Endocrinology. (2019) 160:1421–35. doi: 10.1210/en.2019-00034, PMID: 30998239 PMC6525581

[9] Vandenberg LN Schaeberle CM Rubin BS Sonnenschein C Soto AM. The male mammary gland: A target for the xenoestrogen bisphenol A. Reprod Toxicol. (2013) 37:15–23. doi: 10.1016/j.reprotox.2013.01.002, PMID: 23348055 PMC3998714

[10] Schug TT Janesick A Blumberg B Heindel JJ. Endocrine disrupting chemicals and disease susceptibility. J Steroid Biochem Mol Biol. (2011) 127:204–15. doi: 10.1016/j.jsbmb.2011.08.007, PMID: 21899826 PMC3220783

[11] Satarug S Garrett SH Sens MA Sens DA. Cadmium, environmental exposure, and health outcomes. Environ Health Perspect. (2010) 118:182–90. doi: 10.1289/ehp.0901234, PMID: 20123617 PMC2831915

[12] Balachandar R Bagepally BS Kalahasthi R Haridoss M. Blood lead levels and male reproductive hormones: A systematic review and meta-analysis. Toxicology. (2020) 443:152574. doi: 10.1016/j.tox.2020.152574, PMID: 32860866

[13] Mawia AM Hui S Zhou L Li H Tabassum J Lai C. Inorganic arsenic toxicity and alleviation strategies in rice. J Hazardous Materials. (2021) 408:124751. doi: 10.1016/j.jhazmat.2020.124751, PMID: 33418521

[14] Liu J Kang Y Yin S Song B Wei L Chen L. Zinc oxide nanoparticles induce toxic responses in human neuroblastoma SHSY5Y cells in a size-dependent manner. Int J Nanomedicine. (2017) 12:8085–99. doi: 10.2147/ijn.S149070, PMID: 29138564 PMC5677299

[15] Hu H Fan X Yin Y Guo Q Yang D Wei X. Mechanisms of titanium dioxide nanoparticle-induced oxidative stress and modulation of plasma glucose in mice. Environ Toxicol. (2019) 34:1221–35. doi: 10.1002/tox.22823, PMID: 31298478

[16] Vandenberg LN Maffini MV Sonnenschein C Rubin BS Soto AM. Bisphenol-A and the great divide: A review of controversies in the field of endocrine disruption. Endocrine Rev. (2009) 30:75–95. doi: 10.1210/er.2008-0021, PMID: 19074586 PMC2647705

[17] Kay VR Bloom MS Foster WG. Reproductive and developmental effects of phthalate diesters in males. Crit Rev Toxicol. (2014) 44:467–98. doi: 10.3109/10408444.2013.875983, PMID: 24903855

[18] Darnerud PO. Brominated flame retardants as possible endocrine disrupters. Int J Andrology. (2008) 31:152–60. doi: 10.1111/j.1365-2605.2008.00869.x, PMID: 18315715

[19] Saegusa Y Fujimoto H Woo G-H Inoue K Takahashi M Mitsumori K. Developmental toxicity of brominated flame retardants, tetrabromobisphenol A and 1,2,5,6,9,10-hexabromocyclododecane, in rat offspring after maternal exposure from mid-gestation through lactation. Reprod Toxicol. (2009) 28:456–67. doi: 10.1016/j.reprotox.2009.06.011, PMID: 19577631

[20] Lauby-Secretan B Loomis D Grosse Y Ghissassi FE Bouvard V Benbrahim-Tallaa L. Carcinogenicity of polychlorinated biphenyls and polybrominated biphenyls. Lancet Oncol. (2013) 14:287–8. doi: 10.1016/s1470-2045(13)70104-9, PMID: 23499544

[21] White SS Birnbaum LS. An overview of the effects of dioxins and dioxin-like compounds on vertebrates, as documented in human and ecological epidemiology. J Environ Sci Health Part C. (2009) 27:197–211. doi: 10.1080/10590500903310047, PMID: 19953395 PMC2788749

[22] Cohn BA La Merrill M Krigbaum NY Yeh G Park J-S Zimmermann L. DDT exposure in utero and breast cancer. J Clin Endocrinol Metab. (2015) 100:2865–72. doi: 10.1210/jc.2015-1841, PMID: 26079774 PMC4524999

[23] Rehman S Usman Z Rehman S AlDraihem M Rehman N Rehman I. Endocrine disrupting chemicals and impact on male reproductive health. Trans Andrology Urol. (2018) 7:490–503. doi: 10.21037/tau.2018.05.17, PMID: 30050807 PMC6043754

[24] Giulioni C Maurizi V De Stefano V Polisini G Teoh JY-C Milanese G. The influence of lead exposure on male semen parameters: A systematic review and meta-analysis. Reprod Toxicol. (2023) 118 :108387. doi: 10.1016/j.reprotox.2023.108387, PMID: 37119974

[25] Yucra S Gasco M Rubio J Gonzales GF. Semen quality in Peruvian pesticide applicators: association between urinary organophosphate metabolites and semen parameters. Environ Health. (2008) 7 :59. doi: 10.1186/1476-069x-7-59, PMID: 19014632 PMC2588569

[26] Bellingham M Evans N. IMPACT OF REAL-LIFE ENVIRONMENTAL EXPOSURES ON REPRODUCTION: Biosolids and male reproduction. Reproduction. (2024) 168(2):e240119. doi: 10.1530/rep-24-0119, PMID: 38847770 PMC11286255

[27] Carré J Gatimel N Moreau J Parinaud J Léandri R. Does air pollution play a role in infertility?: a systematic review. Environ Health. (2017) 16(1):82. doi: 10.1186/s12940-017-0291-8, PMID: 28754128 PMC5534122

[28] Dehdashti B Nikaeen M Amin MM Mohammadi F. Health risk assessment of exposure to bisphenol A in polymeric baby bottles. Environ Health Insights. (2023) 17:11786302231151531. doi: 10.1177/11786302231151531, PMID: 36726789 PMC9885033

[29] Meeker JD Stapleton HM. House dust concentrations of organophosphate flame retardants in relation to hormone levels and semen quality parameters. Environ Health Perspect. (2010) 118:318–23. doi: 10.1289/ehp.0901332, PMID: 20194068 PMC2854757

[30] Cheong A Johnson SA Howald EC Ellersieck MR Camacho L Lewis SM. Gene expression and DNA methylation changes in the hypothalamus and hippocampus of adult rats developmentally exposed to bisphenol A or ethinyl estradiol: a CLARITY-BPA consortium study. Epigenetics. (2018) 13:704–20. doi: 10.1080/15592294.2018.1497388, PMID: 30001178 PMC6224219

[31] Sharma R Agarwal A Mohanty G Hamada AJ Gopalan B Willard B. Proteomic analysis of human spermatozoa proteins with oxidative stress. Reprod Biol Endocrinol. (2013) 11:48. doi: 10.1186/1477-7827-11-48, PMID: 23688036 PMC3716960

[32] Harman C Allan IJ Vermeirssen ELM. Calibration and use of the polar organic chemical integrative sampler—a critical review. Environ Toxicol Chem. (2012) 31:2724–38. doi: 10.1002/etc.2011, PMID: 23012256

[33] Brack W Ait-Aissa S Burgess RM Busch W Creusot N Di Paolo C. Effect-directed analysis supporting monitoring of aquatic environments — An in-depth overview. Sci Total Environ. (2016) 544:1073–118. doi: 10.1016/j.scitotenv.2015.11.102, PMID: 26779957

[34] Xu L-C Sun H Chen J-F Bian Q Qian J Song L. Evaluation of androgen receptor transcriptional activities of bisphenol A, octylphenol and nonylphenol *in vitro* . Toxicology. (2005) 216:197–203. doi: 10.1016/j.tox.2005.08.006, PMID: 16169144

[35] Chmelar R Buchanan G Need EF Tilley W Greenberg NM. Androgen receptor coregulators and their involvement in the development and progression of prostate cancer. Int J Cancer. (2006) 120:719–33. doi: 10.1002/ijc.22365, PMID: 17163421

[36] Kuiper GGJM Lemmen JG Carlsson B Corton JC Safe SH van der Saag PT. Interaction of estrogenic chemicals and phytoestrogens with estrogen receptor β. Endocrinology. (1998) 139:4252–63. doi: 10.1210/endo.139.10.6216, PMID: 9751507

[37] Bulayeva NN Watson CS. Xenoestrogen-induced ERK-1 and ERK-2 activation via multiple membrane-initiated signaling pathways. Environ Health Perspect. (2004) 112:1481–7. doi: 10.1289/ehp.7175, PMID: 15531431 PMC1325963

[38] Sun Y Liu W-Z Liu T Feng X Yang N Zhou H-F. Signaling pathway of MAPK/ERK in cell proliferation, differentiation, migration, senescence and apoptosis. J Receptors Signal Transduction. (2015) 35:600–4. doi: 10.3109/10799893.2015.1030412, PMID: 26096166

[39] Filardo EJ Quinn JA Bland KI Frackelton AR. Estrogen-Induced Activation of Erk-1 and Erk-2 Requires the G Protein-Coupled Receptor Homolog, GPR30, and Occurs via Trans-Activation of the Epidermal Growth Factor Receptor through Release of HB-EGF. Mol Endocrinol. (2000) 14:1649–60. doi: 10.1210/mend.14.10.0532, PMID: 11043579

[40] Wozniak AL Bulayeva NN Watson CS. Xenoestrogens at picomolar to nanomolar concentrations trigger membrane estrogen receptor-α–mediated ca 2+ Fluxes and prolactin release in GH3/B6 pituitary tumor cells. Environ Health Perspect. (2005) 113:431–9. doi: 10.1289/ehp.7505, PMID: 15811834 PMC1278483

[41] Ben-Yosef D. Early ionic events in activation of the mammalian egg. Rev Reprod. (1998) 3:96–103. doi: 10.1530/ror.0.0030096, PMID: 9685188

[42] Rahman MS Kwon W-S Pang M-G. Calcium influx and male fertility in the context of the sperm proteome: an update. BioMed Res Int. (2014) 2014:1–13. doi: 10.1155/2014/841615, PMID: 24877140 PMC4022195

[43] Henson MC Chedrese PJ. Endocrine disruption by cadmium, a common environmental toxicant with paradoxical effects on reproduction. Exp Biol Med. (2004) 229:383–92. doi: 10.1177/153537020422900506, PMID: 15096650

[44] Sanderson JT Boerma J Lansbergen GWA van den Berg M. Induction and inhibition of aromatase (CYP19) activity by various classes of pesticides in H295R human adrenocortical carcinoma cells. Toxicol Appl Pharmacol. (2002) 182:44–54. doi: 10.1006/taap.2002.9420, PMID: 12127262

[45] Nebert DW Dalton TP. The role of cytochrome P450 enzymes in endogenous signalling pathways and environmental carcinogenesis. Nat Rev Cancer. (2006) 6:947–60. doi: 10.1038/nrc2015, PMID: 17128211

[46] Zanger UM Schwab M. Cytochrome P450 enzymes in drug metabolism: Regulation of gene expression, enzyme activities, and impact of genetic variation. Pharmacol Ther. (2013) 138:103–41. doi: 10.1016/j.pharmthera.2012.12.007, PMID: 23333322

[47] Valko M Rhodes CJ Moncol J Izakovic M Mazur M. Free radicals, metals and antioxidants in oxidative stress-induced cancer. Chemico-Biological Interact. (2006) 160:1–40. doi: 10.1016/j.cbi.2005.12.009, PMID: 16430879

[48] Hayes JD Strange RC. Glutathione S-transferase polymorphisms and their biological consequences. Pharmacology. (2000) 61:154–66. doi: 10.1159/000028396, PMID: 10971201

[49] Youle RJ van der Bliek AM. Mitochondrial fission, fusion, and stress. Science. (2012) 337:1062–5. doi: 10.1126/science.1219855, PMID: 22936770 PMC4762028

[50] Chen H Chan DC. Mitochondrial dynamics-fusion, fission, movement, and mitophagy-in neurodegenerative diseases. Hum Mol Genet. (2009) 18:R169–76. doi: 10.1093/hmg/ddp326, PMID: 19808793 PMC2758711

[51] Narendra D Tanaka A Suen D-F Youle RJ. Parkin is recruited selectively to impaired mitochondria and promotes their autophagy. J Cell Biol. (2008) 183:795–803. doi: 10.1083/jcb.200809125, PMID: 19029340 PMC2592826

[52] Hüttemann M Lee I Grossman LI Doan JW Sanderson TH. Phosphorylation of Mammalian Cytochrome c and Cytochrome c Oxidase in the Regulation of Cell Destiny: Respiration, Apoptosis, and Human Disease. Mitochondrial Oxid Phosphorylation. (2012) 748:237–64. doi: 10.1007/978-1-4614-3573-0_10, PMID: 22729861 PMC3727645

[53] Michael P. Murphy: How mitochondria produce reactive oxygen species. Biochem J. (2008) 417:1–13. doi: 10.1042/bj20081386, PMID: 19061483 PMC2605959

[54] Kang KW Lee SJ Kim SG. Molecular mechanism of nrf2 activation by oxidative stress. Antioxidants Redox Signaling. (2005) 7:1664–73. doi: 10.1089/ars.2005.7.1664, PMID: 16356128

[55] Brookes PS Yoon Y Robotham JL Anders MW Sheu S-S. Calcium, ATP, and ROS: a mitochondrial love-hate triangle. Am J Physiology-Cell Physiol. (2004) 287:C817–33. doi: 10.1152/ajpcell.00139.2004, PMID: 15355853

[56] Vander Heiden MG Cantley LC Thompson CB. Understanding the warburg effect: the metabolic requirements of cell proliferation. Science. (2009) 324:1029–33. doi: 10.1126/science.1160809, PMID: 19460998 PMC2849637

[57] Rokhlin OW Scheinker VS Taghiyev AF Bumcrot D Glover RA Cohen MB. MicroRNA-34 mediates AR-dependent p53-induced apoptosis in prostate cancer. Cancer Biol Ther. (2014) 7:1288–96. doi: 10.4161/cbt.7.8.6284, PMID: 18497571

[58] Lu C Liao Z Cai M Zhang G. MicroRNA-320a downregulation mediates human liver cancer cell proliferation through the Wnt/β-catenin signaling pathway. Oncol Lett. (2017) 13:573–8. doi: 10.3892/ol.2016.5479, PMID: 28356931 PMC5351300

[59] Litton C Benny P Lambertini L Ma Y Riel J Weingrill R. Epigenetic changes in the HTR8 and 3A-sub E placental cell lines exposed to bisphenol A and benzyl butyl phthalate. Toxics 12(9). (2024) 12(9):659. doi: 10.3390/toxics12090659, PMID: 39330587 PMC11435974

[60] Wang X Yang Y Guo X Sampson ER Hsu C-L Tsai M-Y. Suppression of androgen receptor transactivation by pyk2 via interaction and phosphorylation of the ARA55 coregulator. J Biol Chem. (2002) 277:15426–31. doi: 10.1074/jbc.M111218200, PMID: 11856738

[61] Maatouk DM Natarajan A Shibata Y Song L Crawford GE Ohler U. Genome-wide identification of regulatory elements in Sertoli cells. Development. (2017) 144:720–30. doi: 10.1242/dev.142554, PMID: 28087634 PMC5312035

[62] Walker DM Gore AC. Transgenerational neuroendocrine disruption of reproduction. Nat Rev Endocrinol. (2011) 7:197–207. doi: 10.1038/nrendo.2010.215, PMID: 21263448 PMC3976559

[63] Xavier MJ Roman SD Aitken RJ Nixon B. Transgenerational inheritance: how impacts to the epigenetic and genetic information of parents affect offspring health. Hum Reprod Update. (2019) 25:518–40. doi: 10.1093/humupd/dmz017, PMID: 31374565

[64] Meeker JD Calafat AM Hauser R. Urinary bisphenol A concentrations in relation to serum thyroid and reproductive hormone levels in men from an infertility clinic. Environ Sci Technol. (2009) 44:1458–63. doi: 10.1021/es9028292, PMID: 20030380 PMC2823133

[65] Miri M Nazarzadeh M Alahabadi A Ehrampoush MH Rad A Lotfi MH. Air pollution and telomere length in adults: A systematic review and meta-analysis of observational studies. Environ pollut. (2019) 244:636–47. doi: 10.1016/j.envpol.2018.09.130, PMID: 30384069

[66] Hauser R Meeker JD Singh NP Silva MJ Ryan L Duty S. DNA damage in human sperm is related to urinary levels of phthalate monoester and oxidative metabolites. Hum Reprod. (2007) 22:688–95. doi: 10.1093/humrep/del428, PMID: 17090632

[67] Aitken RJ De Iuliis GN. On the possible origins of DNA damage in human spermatozoa. Mol Hum Reprod. (2009) 16:3–13. doi: 10.1093/molehr/gap059, PMID: 19648152

[68] Smith LB Walker WH. The regulation of spermatogenesis by androgens. Semin Cell Dev Biol. (2014) 30:2–13. doi: 10.1016/j.semcdb.2014.02.012, PMID: 24598768 PMC4043871

[69] Martino-Andrade AJ Chahoud I. Reproductive toxicity of phthalate esters. Mol Nutr Food Res. (2010) 54:148–57. doi: 10.1002/mnfr.200800312, PMID: 19760678

[70] Swan SH Kruse RL Liu F Barr DB Drobnis EZ Redmon JB. Semen quality in relation to biomarkers of pesticide exposure. Environ Health Perspect. (2003) 111:1478–84. doi: 10.1289/ehp.6417, PMID: 12948887 PMC1241650

[71] Thurston SW Mendiola J Bellamy AR Levine H Wang C Sparks A. Phthalate exposure and semen quality in fertile US men. Andrology. (2016) 4:632–8. doi: 10.1111/andr.12124, PMID: 26601918 PMC4879116

[72] Mendiola J Jørgensen N Andersson A-M Calafat AM Ye X Redmon JB. Are environmental levels of bisphenol A associated with reproductive function in fertile men? Environ Health Perspect. (2010) 118:1286–91. doi: 10.1289/ehp.1002037, PMID: 20494855 PMC2944091

[73] Rodprasert W Toppari J Virtanen HE. Environmental toxicants and male fertility. Best Pract Res Clin Obstetrics Gynaecology. (2023) 86:102298. doi: 10.1016/j.bpobgyn.2022.102298, PMID: 36623980

[74] Bloom MS Whitcomb BW Chen Z Ye A Kannan K Buck Louis GM. Associations between urinary phthalate concentrations and semen quality parameters in a general population. Hum Reprod. (2015) 30:2645–57. doi: 10.1093/humrep/dev219, PMID: 26350610 PMC4605371

[75] Agarwal A Saleh RA. Role of oxidants in male infertility: rationale, significance, and treatment. Urologic Clinics North America. (2002) 29:817–27. doi: 10.1016/s0094-0143(02)00081-2, PMID: 12516754

[76] Khosrowbeygi A Zarghami N. Levels of oxidative stress biomarkers in seminal plasma and their relationship with seminal parameters. BMC Clin Pathol. (2007) 7 :6. doi: 10.1186/1472-6890-7-6, PMID: 17540046 PMC1906821

[77] Ahmad R Gautam AK Verma Y Sedha S Kumar S. Effects of in *utero* di-butyl phthalate and butyl benzyl phthalate exposure on offspring development and male reproduction of rat. Environ Sci pollut Res Int. (2014) 21:3156–65. doi: 10.1007/s11356-013-2281-x, PMID: 24213843

[78] Amaral A Ramalho-Santos J St John JC. The expression of polymerase gamma and mitochondrial transcription factor A and the regulation of mitochondrial DNA content in mature human sperm. Hum Reprod. (2007) 22:1585–96. doi: 10.1093/humrep/dem030, PMID: 17339235

[79] Myers JP Zoeller RT Welshons WV vom Saal FS Soto AM Shioda T. Hormones and endocrine-disrupting chemicals: low-dose effects and nonmonotonic dose responses. Endocrine Rev. (2012) 33:378–455. doi: 10.1210/er.2011-1050, PMID: 22419778 PMC3365860

[80] Bonde JP Toft G Rylander L Rignell-Hydbom A Giwercman A Spano M. Fertility and markers of male reproductive function in inuit and european populations spanning large contrasts in blood levels of persistent organochlorines. Environ Health Perspect. (2008) 116:269–77. doi: 10.1289/ehp.10700, PMID: 18335090 PMC2265036

[81] Ji Y Tian Y Pan Y Sheng N Dai H Fan X. Exposure and potential risks of thirteen endocrine- disrupting chemicals in pharmaceuticals and personal care products for breastfed infants in China. Environ Int. (2024) 192:109032. doi: 10.1016/j.envint.2024.109032, PMID: 39317008

[82] Takalani NB Monageng EM Mohlala K Monsees TK Henkel R Opuwari CS. Role of oxidative stress in male infertility. Reprod Fertil. (2023) 4(3):e230024. doi: 10.1530/RAF-23-0024, PMID: 37276172 PMC10388648

[83] Skinner MK Manikkam M Guerrero-Bosagna C. Epigenetic transgenerational actions of endocrine disruptors. Reprod Toxicol. (2011) 31:337–43. doi: 10.1016/j.reprotox.2010.10.012, PMID: 21055462 PMC3068236

[84] Anway MD Cupp AS Uzumcu M Skinner MK. Epigenetic transgenerational actions of endocrine disruptors and male fertility. Science. (2005) 308:1466–9. doi: 10.1126/science.1108190, PMID: 15933200 PMC11423801

[85] Susiarjo M Hassold TJ Freeman E Hunt PA. Bisphenol A exposure *in utero* disrupts early oogenesis in the mouse. PloS Genet. (2007) 3(1):e5. doi: 10.1371/journal.pgen.0030005, PMID: 17222059 PMC1781485

[86] Singh S Li SS-L. Epigenetic effects of environmental chemicals bisphenol A and phthalates. Int J Mol Sci. (2012) 13:10143–53. doi: 10.3390/ijms130810143, PMID: 22949852 PMC3431850

[87] Cheung HH Lee TL Davis AJ Taft DH Rennert OM Chan WY. Genome-wide DNA methylation profiling reveals novel epigenetically regulated genes and non-coding RNAs in human testicular cancer. Br J Cancer. (2010) 102:419–27. doi: 10.1038/sj.bjc.6605505, PMID: 20051947 PMC2816664

[88] von Meyenn F Iurlaro M Habibi E Ning Q. Liu A Salehzadeh-Yazdi F Santos. : impairment of DNA methylation maintenance is the main cause of global demethylation in naive embryonic stem cells. Mol Cell. (2016) 62:848–61. doi: 10.1016/j.molcel.2016.04.025, PMID: 27237052 PMC4914828

[89] Li X Lu Z Du X Ye Y Zhu J Li Y. Prenatal cadmium exposure has inter-generational adverse effects on Sertoli cells through the follicle-stimulating hormone receptor pathway. Reproduction. (2023) 166:271–84. doi: 10.1530/rep-23-0070, PMID: 37590121 PMC10502957

[90] Lite C Raja GL Juliet M Sridhar VV Subhashree KD Kumar P. *In utero* exposure to endocrine-disrupting chemicals, maternal factors and alterations in the epigenetic landscape underlying later-life health effects. Environ Toxicol Pharmacol. (2022) 89:103779. doi: 10.1016/j.etap.2021.103779, PMID: 34843942

[91] Stuppia L Franzago M Ballerini P Gatta V Antonucci I. Epigenetics and male reproduction: the consequences of paternal lifestyle on fertility, embryo development, and children lifetime health. Clin Epigenet. (2015) 7:120. doi: 10.1186/s13148-015-0155-4, PMID: 26566402 PMC4642754

[92] Bromer JG Zhou Y Taylor MB Doherty L Taylor HS. Bisphenol-A exposure in *utero* leads to epigenetic alterations in the developmental programming of uterine estrogen response. FASEB J. (2010) 24:2273–80. doi: 10.1096/fj.09-140533, PMID: 20181937 PMC3230522

[93] Cariati F Carbone L Conforti A Bagnulo F Peluso SR Carotenuto C. Bisphenol A-induced epigenetic changes and its effects on the male reproductive system. Front Endocrinol (Lausanne). (2020) 11:453. doi: 10.3389/fendo.2020.00453, PMID: 32849263 PMC7406566

[94] Wu S Zhu J Li Y Lin T Gan L Yuan X. Dynamic epigenetic changes involved in testicular toxicity induced by di-2-(Ethylhexyl) phthalate in mice. Basic Clin Pharmacol Toxicol. (2010) 106:118–23. doi: 10.1111/j.1742-7843.2009.00483.x, PMID: 19912166

[95] Cribbs AP Terlecki-Zaniewicz S Philpott M Baardman J Ahern D Lindow M. Histone H3K27me3 demethylases regulate human Th17 cell development and effector functions by impacting on metabolism. Proc Natl Acad Sci. (2020) 117:6056–66. doi: 10.1073/pnas.1919893117, PMID: 32123118 PMC7084125

[96] Martinez-Pacheco M Hidalgo-Miranda A Romero-Cordoba S Valverde M Rojas E. MRNA and miRNA expression patterns associated to pathways linked to metal mixture health effects. Gene. (2014) 533:508–14. doi: 10.1016/j.gene.2013.09.049, PMID: 24080485

[97] Hayashi M Kawaguchi T Durcova-Hills G Imai H. Generation of germ cells from pluripotent stem cells in mammals. Reprod Med Biol. (2017) 17:107–14. doi: 10.1002/rmb2.12077, PMID: 29692667 PMC5902460

[98] Liu W-M Pang RTK Chiu PCN Wong BPC Lao K Lee K-F. Sperm-borne microRNA-34c is required for the first cleavage division in mouse. Proc Natl Acad Sci. (2011) 109:490–4. doi: 10.1073/pnas.1110368109, PMID: 22203953 PMC3258645

[99] Slotkin RK Martienssen R. Transposable elements and the epigenetic regulation of the genome. Nat Rev Genet. (2007) 8:272–85. doi: 10.1038/nrg2072, PMID: 17363976

[100] Shioda T Manikkam M Tracey R Guerrero-Bosagna C Skinner MK. Plastics derived endocrine disruptors (BPA, DEHP and DBP) induce epigenetic transgenerational inheritance of obesity, reproductive disease and sperm epimutations. PloS One. (2013) 8(1):e55387. doi: 10.1371/journal.pone.0055387, PMID: 23359474 PMC3554682

[101] Guerrero-Bosagna C Skinner MK. Environmentally induced epigenetic transgenerational inheritance of male infertility. Curr Opin Genet Dev. (2014) 26:79–88. doi: 10.1016/j.gde.2014.06.005, PMID: 25104619 PMC4252707

[102] Nilsson EE Skinner MK. Environmentally induced epigenetic transgenerational inheritance of disease susceptibility. Trans Res. (2015) 165:12–7. doi: 10.1016/j.trsl.2014.02.003, PMID: 24657180 PMC4148471

[103] Nilsson E King SE McBirney M Kubsad D Pappalardo M Beck D. Vinclozolin induced epigenetic transgenerational inheritance of pathologies and sperm epimutation biomarkers for specific diseases. PloS One. (2018) 13:e0202662. doi: 10.1371/journal.pone.0202662, PMID: 30157260 PMC6114855

[104] Alavian-Ghavanini A Ruegg J. Understanding epigenetic effects of endocrine disrupting chemicals: from mechanisms to novel test methods. Basic Clin Pharmacol Toxicol. (2018) 122:38–45. doi: 10.1111/bcpt.12878, PMID: 28842957

[105] Palak E Lebiedzinska W Anisimowicz S Sztachelska M Pierzynski P Wiczkowski W. The association between bisphenol A, steroid hormones, and selected microRNAs levels in seminal plasma of men with infertility. J Clin Med. (2021) 10(24):5945. doi: 10.3390/jcm10245945, PMID: 34945242 PMC8703400

[106] Xiong YW Zhu HL Zhang J Geng H Tan LL Zheng XM. Multigenerational paternal obesity enhances the susceptibility to male subfertility in offspring via Wt1 N6-methyladenosine modification. Nat Commun. (2024) 15:1353. doi: 10.1038/s41467-024-45675-4, PMID: 38355624 PMC10866985

[107] Walke G Gaurkar SS Prasad R Lohakare T Wanjari M. The impact of oxidative stress on male reproductive function: exploring the role of antioxidant supplementation. Cureus. (2023) 15:e42583. doi: 10.7759/cureus.42583, PMID: 37641770 PMC10460465

[108] Klibaner-Schiff E Simonin EM Akdis CA Cheong A Johnson MM Karagas MR. Environmental exposures influence multigenerational epigenetic transmission. Clin Epigenet. (2024) 16:145. doi: 10.1186/s13148-024-01762-3, PMID: 39420431 PMC11487774

[109] Zhu J Guo S Cao J Zhao H Ma Y Zou H. Epigenetic modifications are involved in transgenerational inheritance of cadmium reproductive toxicity in mouse oocytes. Int J Mol Sci 25(20). (2024) 25(20):10996. doi: 10.3390/ijms252010996, PMID: 39456778 PMC11507422

[110] King SE Skinner MK. Epigenetic transgenerational inheritance of obesity susceptibility. Trends Endocrinol Metab. (2020) 31:478–94. doi: 10.1016/j.tem.2020.02.009, PMID: 32521235 PMC8260009

[111] Serrano JB Tabeling NC de Winter-Korver CM van Daalen SKM van Pelt AMM Mulder CL. Sperm DNA methylation is predominantly stable in mice offspring born after transplantation of long-term cultured spermatogonial stem cells. Clin Epigenetics 15(1). (2023) 15(1):58. doi: 10.1186/s13148-023-01469-x, PMID: 37029425 PMC10080964

[112] Anway MD Leathers C Skinner MK. Endocrine disruptor vinclozolin induced epigenetic transgenerational adult-onset disease. Endocrinology. (2006) 147:5515–23. doi: 10.1210/en.2006-0640, PMID: 16973726 PMC5940332

[113] Tao Y Li Z Yang Y Jiao Y Qu J Wang Y. Effects of common environmental endocrine-disrupting chemicals on zebrafish behavior. Water Res. (2022) 208:117826. doi: 10.1016/j.watres.2021.117826, PMID: 34785404

[114] Ho S-M Cheong A Adgent MA Veevers J Suen AA Tam NNC. Environmental factors, epigenetics, and developmental origin of reproductive disorders. Reprod Toxicol. (2017) 68:85–104. doi: 10.1016/j.reprotox.2016.07.011, PMID: 27421580 PMC5233640

[115] Nelson VR Heaney JD Tesar PJ Davidson NO Nadeau JH. Transgenerational epigenetic effects of the Apobec1 cytidine deaminase deficiency on testicular germ cell tumor susceptibility and embryonic viability. Proc Natl Acad Sci U.S.A. (2012) 109:E2766–73. doi: 10.1073/pnas.1207169109, PMID: 22923694 PMC3478648

[116] Gore AC Thompson LM Bell M Mennigen JA. Transgenerational effects of polychlorinated biphenyls: 2. Hypothalamic Gene Expression ratsdagger. Biol Reprod. (2021) 105:690–704. doi: 10.1093/biolre/ioab066, PMID: 33824955 PMC8444700

[117] Li DK Zhou Z Miao M He Y Wang J Ferber J. Urine bisphenol-A (BPA) level in relation to semen quality. Fertil Steril. (2011) 95:625–30 e1-4. doi: 10.1016/j.fertnstert.2010.09.026, PMID: 21035116

[118] Vojta A Dobrinić P Tadić V Bočkor L Korać P Julg B. Repurposing the CRISPR-Cas9 system for targeted DNA methylation. Nucleic Acids Res. (2016) 44:5615–28. doi: 10.1093/nar/gkw159, PMID: 26969735 PMC4937303

[119] Zhang N Wang Y Chen Z Ren J Rehman A Ahmad DW. Single-cell transcriptome analysis of Bisphenol A exposure reveals the key roles of the testicular microenvironment in male reproduction. Biomedicine Pharmacotherapy 145. (2022) 145: 112449. doi: 10.1016/j.biopha.2021.112449, PMID: 34808557

[120] LaDou J. The asbestos cancer epidemic. Environ Health Perspect. (2004) 112:285–90. doi: 10.1289/ehp.6704, PMID: 14998741 PMC1241855

[121] Bhuiyan MAR Wang L Shaid A Shanks RA Ding J. Advances and applications of chemical protective clothing system. J Ind Textiles. (2018) 49:97–138. doi: 10.1177/1528083718779426

[122] Jensen TK Bonde JP Joffe M. The influence of occupational exposure on male reproductive function. Occup Med. (2006) 56:544–53. doi: 10.1093/occmed/kql116, PMID: 17151390

[123] Leigh JP. Economic burden of occupational injury and illness in the United States. Milbank Q. (2011) 89:728–72. doi: 10.1111/j.1468-0009.2011.00648.x, PMID: 22188353 PMC3250639

[124] MacFarlane E Carey R Keegel T El-Zaemay S Fritschi L. Dermal exposure associated with occupational end use of pesticides and the role of protective measures. Saf Health at Work. (2013) 4:136–41. doi: 10.1016/j.shaw.2013.07.004, PMID: 24106643 PMC3791087

[125] Ye M Beach J Martin J Senthilselvan A. Occupational pesticide exposures and respiratory health. Int J Environ Res Public Health. (2013) 10:6442–71. doi: 10.3390/ijerph10126442, PMID: 24287863 PMC3881124

[126] Rupa DS Reddy PP Reddi OS. Reproductive performance in population exposed to pesticides in cotton fields in India. Environ Res. (1991) 55:123–8. doi: 10.1016/s0013-9351(05)80168-9, PMID: 1868815

[127] Benotti MJ Snyder SA. Endocrine disruptors and pharmaceuticals: implications for water sustainability. Water Sci Technol. (2010) 61:145–54. doi: 10.2166/wst.2010.791, PMID: 20057100

[128] Deblonde T Cossu-Leguille C Hartemann P. Emerging pollutants in wastewater: A review of the literature. Int J Hygiene Environ Health. (2011) 214:442–8. doi: 10.1016/j.ijheh.2011.08.002, PMID: 21885335

[129] orld Health Organization. Committee. Guidelines for drinking-water quality: fourth edition incorporating the first addendum. (2017). Bookshelf ID: NBK442376, PMID: 28759192

[130] Meeker JD Ferguson KK. Relationship between urinary phthalate and bisphenol A concentrations and serum thyroid measures in U.S. Adults and adolescents from the national health and nutrition examination survey (NHANES) 2007–2008. Environ Health Perspect. (2011) 119:1396–402. doi: 10.1289/ehp.1103582, PMID: 21749963 PMC3230451

[131] Rudel RA Gray JM Engel CL Rawsthorne TW Dodson RE Ackerman JM. Food packaging and bisphenol A and bis(2-ethyhexyl) phthalate exposure: findings from a dietary intervention. Environ Health Perspect. (2011) 119:914–20. doi: 10.1289/ehp.1003170, PMID: 21450549 PMC3223004

[132] Saini A Okeme JO Mark Parnis J McQueen RH Diamond ML. From air to clothing: characterizing the accumulation of semi-volatile organic compounds to fabrics in indoor environments. Indoor Air. (2017) 27:631–41. doi: 10.1111/ina.12328, PMID: 27555567

[133] Calafat AM Needham LL. Factors affecting the evaluation of biomonitoring data for human exposure assessment. Int J Andrology. (2007) 31:139–43. doi: 10.1111/j.1365-2605.2007.00826.x, PMID: 17971164

[134] Balawender K Orkisz S. The impact of selected modifiable lifestyle factors on male fertility in the modern world. Cent Eur J Urol. (2020) 73:563–8. doi: 10.5173/ceju.2020.1975, PMID: 33552585 PMC7848840

[135] Wu X Lu M Yun D Gao S Chen S Hu L. Single-cell ATAC-Seq reveals cell type-specific transcriptional regulation and unique chromatin accessibility in human spermatogenesis. Hum Mol Genet. (2022) 31:321–33. doi: 10.1093/hmg/ddab006, PMID: 33438010

[136] Lau BT Almeda A Schauer M McNamara M Bai X Meng Q. Single-molecule methylation profiles of cell-free DNA in cancer with nanopore sequencing. Genome Med. (2023) 15(1):33. doi: 10.1186/s13073-023-01178-3, PMID: 37138315 PMC10155347

[137] Jiang F Jiang Y Zhi H Dong Y Li H Ma S. Artificial intelligence in healthcare: past, present and future. Stroke Vasc Neurol. (2017) 2:230–43. doi: 10.1136/svn-2017-000101, PMID: 29507784 PMC5829945

[138] Hosseini M Khalafiyan A Zare M Karimzadeh H Bahrami B Hammami B. Sperm epigenetics and male infertility: unraveling the molecular puzzle. Hum Genomics. (2024) 18(1):57. doi: 10.1186/s40246-024-00626-4, PMID: 38835100 PMC11149391

[139] Liang P Xu Y Zhang X Ding C Huang R Zhang Z. CRISPR/Cas9-mediated gene editing in human tripronuclear zygotes. Protein Cell. (2015) 6:363–72. doi: 10.1007/s13238-015-0153-5, PMID: 25894090 PMC4417674

[140] Dangwal S Thum T. microRNA therapeutics in cardiovascular disease models. Annu Rev Pharmacol Toxicol. (2014) 54:185–203. doi: 10.1146/annurev-pharmtox-011613-135957, PMID: 24111539

[141] Giassetti MI Ciccarelli M Oatley JM. Spermatogonial stem cell transplantation: insights and outlook for domestic animals. Annu Rev Anim Biosci. (2019) 7:385–401. doi: 10.1146/annurev-animal-020518-115239, PMID: 30762440

[142] Jabari A Sadighi Gilani MA Koruji M Gholami K Mohsenzadeh M Rastegar T. Three-dimensional co-culture of human spermatogonial stem cells with Sertoli cells in soft agar culture system supplemented by growth factors and Laminin. Acta Histochemica. (2020) 122(5):151572. doi: 10.1016/j.acthis.2020.151572, PMID: 32622422

[143] Lim JJ Dutta M Dempsey JL Lehmler H-J MacDonald J Bammler T. Neonatal exposure to BPA, BDE-99, and PCB produces persistent changes in hepatic transcriptome associated with gut dysbiosis in adult mouse livers. Toxicological Sci. (2021) 184:83–103. doi: 10.1093/toxsci/kfab104, PMID: 34453844 PMC8557404

[144] Van Cauwenbergh O Di Serafino A Tytgat J Soubry A. Transgenerational epigenetic effects from male exposure to endocrine-disrupting compounds: a systematic review on research in mammals. Clin Epigenetics 12(1). (2020) 12(1):65. doi: 10.1186/s13148-020-00845-1, PMID: 32398147 PMC7218615

[145] Liu C Ma J Amos CI. Bayesian variable selection for hierarchical gene–environment and gene–gene interactions. Hum Genet. (2014) 134:23–36. doi: 10.1007/s00439-014-1478-5, PMID: 25154630 PMC4282989

[146] Scalisi EM Pecoraro R Scalisi A Dragotto J Bracchitta G Zimbone M. Susceptibility of human spermatozoa to titanium dioxide nanoparticles: evaluation of DNA damage and biomarkers. Life. (2024) 14(11):1455. doi: 10.3390/life14111455, PMID: 39598253 PMC11595473

[147] Tohyama C Honda Y. Challenges in health risk assessment of multiple chemical exposures in epidemiological studies. Environ Health Prev Med. (2024) 29:6–6. doi: 10.1265/ehpm.23-00312, PMID: 38325855 PMC10898861

[148] Pelkonen O Bennekou SH Crivellente F Terron A Hernandez AF. Integration of epidemiological findings with mechanistic evidence in regulatory pesticide risk assessment: EFSA experiences. Arch Toxicol. (2019) 93:1779–88. doi: 10.1007/s00204-019-02467-w, PMID: 31053889

[149] Fuller R Landrigan PJ Balakrishnan K Bathan G Bose-O’Reilly S Brauer M. Pollution and health: a progress update. Lancet Planetary Health. (2022) 6:e535–47. doi: 10.1016/s2542-5196(22)00090-0, PMID: 35594895 PMC11995256

[150] Bornman MS Aneck-Hahn NH de Jager C Wagenaar GM Bouwman H Barnhoorn IEJ. Endocrine disruptors and health effects in africa: A call for action. Environ Health Perspect. (2017) 125(8):085005. doi: 10.1289/ehp1774, PMID: 28935616 PMC5783641

